# Colorless-to-colorful switching electrochromic polyimides with very high contrast ratio

**DOI:** 10.1038/s41467-019-09054-8

**Published:** 2019-03-18

**Authors:** Qiang Zhang, Chou-Yi Tsai, Lain-Jong Li, Der-Jang Liaw

**Affiliations:** 1 Department of Chemical Engineering, National Taiwan University of Science and Technology, 10607 Taipei, Taiwan; 2 School of Materials Science and Engineering, University of New South Wales, 2052 NSW, Australia

## Abstract

Colorless-to-colorful switching electrochromic polymers with very high contrast ratio are unattainable and attractive for the applications of smart wearable electronics. Here we report a facile strategy in developing colorless-to-colorful switching electrochromic polyimides by incorporating with alicyclic nonlinear, twisted structures and adjusted conjugated electrochromophores, which minimize the charge transfer complex formation. It is noted that, by controlling the conjugation length of electrochromophore, the colorless-to-black switching electrochromic polymer film (PI-1a) exhibites an ultrahigh integrated contrast ratio up to 91.4% from 380 to 780 nm, especially up to 96.8% at 798 nm. In addition, PI-1a film with asymmetric structure also demonstrates fast electrochemical and electrochromic behaviors (a switching and bleaching time of 1.3 s and 1.1 s, respectively) due to the loose chain stacking, which provides more pathways for the penetration of counterion. Moreover, the colorless-to-black EC device based on PI-1a reveals an overall integrated contrast ratio up to 80%.

## Abstract (web summary)

Electrochromic polymers (ECPs) receive great attention due to their facile colour tunablility, however colourless-to-black ECPs with high contrast ratio are still unattainable. Here the authors develop high contrast colourless-to-black switching polyimides by following specific molecular design criteria.

## Introduction

Non-emissive electrochromic polymers (ECPs) received great attention recent decades due to their low-power consumption, facile color tunablility through molecular design^1^, high processability of highly flexible and rollable displays compared to their inorganic counterparts^2–5^. In developing EC display panels, it needs to include non-emissive chromophores as pixels or superimposed to recreate other different desirable colors^3^, which requires precise control of the colors displayed in terms of their hue, saturation, intensity, and their brightness. However, the colorless-to-colorful ECPs, especially the colorless-to-black ECPs with ultrahigh contrast ratio over the entire visible region, are still unattainable due to the extreme difficulties in design of completely reverse absorptions (transmittance) in transmissive and colored states^5,6^. In addition, the superposition of several desirable colors by color-mixing theory^7^ even deepens the color of EC device in transmissive state. Therefore, in spite of the efforts in developing various colorful ECPs (i.e., black ECPs)^1–11^, both cathodically and anodically coloring ECPs still showed low transmittance in their transmissive states^8–11^. For instance, Reynolds et al.^3,5,6^ initiated the development of black-to-transmissive ECPs through donor-acceptor design, however, weak absorption of these fully oxidized ECP films^5^ in the visible region still existed. In addition, another strategy was developed by Lee et al. and Beverina et al., transmissive-to-black ECPs were obtained by incorporating multichromophores, which had the advantages of superposition of different absorptions from different electrochromophores^8–11^. But it was difficult to control over the absolute redox potential in some cases at the respective electrodes^11^ and the long conjugation also revealed low transmittance in transmissive states^8–11^. Moreover, thick viologen films could be used as colorless-to-black/gray EC materials, however, the thick film exhibited low transmittance in neutral states^12^. Therefore, the development of completely colorless ECP, especially in the field of colorless-to-black ECP, is still remained the most challengeable problem in the field of ECPs up to date.

For decades, triarylamine (TAA) and its derivatives have been used for wide applications in optoelectronic devices^13–17^. In the field of ECPs, TAA showed great advantages because most of their absorptions located at UV region in neutral state^18–20^, which enabled the development of completely colorless ECP films. In addition, TAAs exhibited tunable colors in oxidized states by adjusting conjugation length, which facilitated the design of various colors (including black stage)^18–23^. Therefore, TAA could be considered as the most promising candidates for the colorless-to-colorful ECPs (especially the colorless-to-black ECPs with ultrahigh contrast ratio). On the other hand, based on our previous report^24^, transparent-to-colorful EC polyimides could be developed due to the conjugation break by the imide groups along the main chain. However, the charge transfer complex (CTC) formation in polyimides always generated yellow or brown colors in polyimide films^25^, which may hinder the development of colorless polyimide films.

In this study, we introduced a facile strategy that colorless-to-colorful ECPs, exemplified by PI-1a (colorless-to-black) and PI-2a (colorless-to-blue), could be achieved through preparing polyimides with specific molecular designs. In particular, polyimides were polymerized by twisted^26^ TAA diamines and alicyclic nonlinear diimides, which significantly reduced the CTC formation and enhanced the visible transparency in neutral state. On the other hand, the EC colors were tuned by adjusting the conjugations within an adjusted length, enabling various colorful electrochromism (including black stage) in oxidized states and colorless appearance in neutral states^19^. In addition, EC behavior of PI-1a with asymmetric pendant groups is faster than that of PI-2a, which was related with the looser chain stacking and resulting faster penetration of counterion^19,27^. The facile strategy in developing colorless-to-colorful ECPs with ultrahigh contrast could be especially practical in EC displays and optical areas.

## Results

### Synthesis and characterization

All the chemical structures of polyimides were shown in Figs. 1 and 2. They were prepared via coupling reaction and polycondensation, the synthetic routes (see Supplementary Figure 1, Supplementary Figure 6 and Supplementary Figure 8) and characterizations (see Supplementary Figures 2–5, Supplementary Figure 7, Supplementary Figures 9–10) were described in Supplementary Methods.

**Fig. 1:**
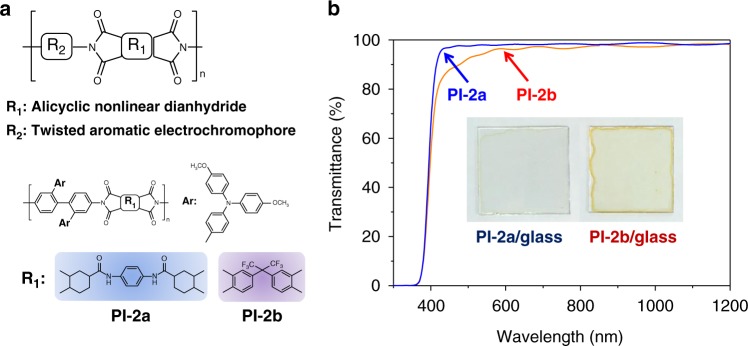
Polyimide structures and comparison of their transmittance spectra. **a** General chemical formula of transparent polyimides incorporation of nonlinear alicyclic diimide and twisted TAA-containing diamine. **b** Transmittance spectra of PI-2a and PI-2a films

**Fig. 2:**
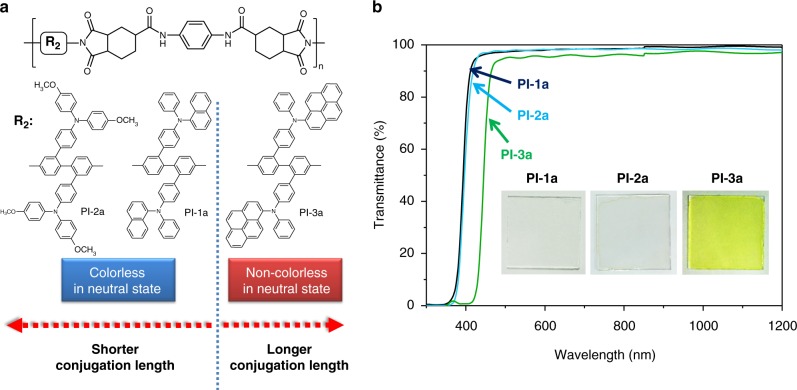
EC polyimide structures and their transmittance spectra. **a** Chemical structures of PI-1a, PI-2a, and PI-3a. **b** Transmittance spectra of PI-1a, PI-2a and PI-3a films on ITO glass. Insert photographs show the colors of PI-1a, PI-2a, and PI-3a films

### Facile strategy of colorless-to-colorful ECPs

We show that the facile strategy in developing colorless-to-colorful EC polyimides is by incorporating alicyclic nonlinear diimide and twisted TAA-containing diamine (see Fig. 1a), exemplified by colorless-to-black (PI-1a, see Fig. 2a) and colorless-to-blue (PI-2a, see Fig. 2a) switching ECPs in this study.

### Superior transmittance

Figure 1b shows the transmittance spectra of polyimide films (PI-2a and PI-2b) using alicyclic nonlinear HTA-PPD and 4,4′-(hexafluoroisopropylidene) diphthalic anhydride (6FDA, a widely used dianhydride for preparation of transparent polyimides)^28^. PI-2a film was colorless and transparent while PI-2b film was slightly red-brown (see Supplementary Figure 11a), the enhancement of PI-2a film transmittance spectra in the range of 400–600 nm (see Fig. 1b) could be attributed to the inhibited CTC, resulted from the nonlinear (*cis, cis*)-alicyclic moieties of HTA-PPD (characterized in Supplementary Figure 4)^29^. The photoluminescence (PL) excitation maps (PLE) showed an intensive PL of PI-2a film (see Supplementary Figure 11c) and a quenched PL of PI-2b film (see Supplementary Figure 11d), indicative of the CTC absence of PI-2a film^30,31^, which was attributed to the nonlinear cyclohexyl moiety of HTA-PPD^32^.

### EC behavior of PI-1a and PI-2a films

Three kinds of TAA derivatives with different conjugation lengths ((1) (*N*-phenyl-*N*-phenyl)naphthalen-1-amine, (2) bis(4-methoxy-*N*-phenyl)aniline, and (3) (*N*-phenyl-*N*-phenyl)pyrene-1-amine, as shown in Fig. 2a) were bonded to the *meta*-positions of diphenyldiamine as pendant groups to form noncoplanar structures. As revealed in Fig. 2a, b, the polyimide film became yellow when the conjugation length of pendant group extended to *N*-phenyl-*N*-phenyl)pyrene-1-amine, while the PIs with shorter conjugations were colorless in neutral state. In particular, Fig. 2b showed the photographs of colorless PI-1a and PI-2a films in neutral state. The electronic properties of PI-1a and PI-2a were summarized in Supplementary Table 1.

**Fig. 3:**
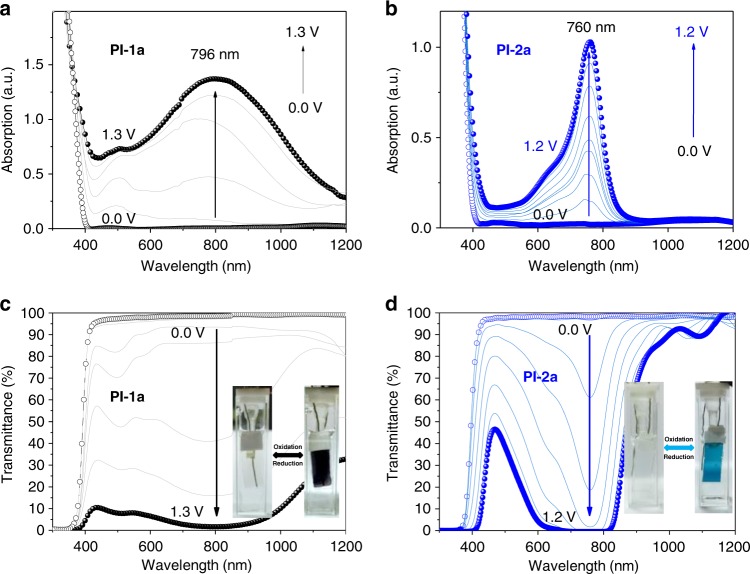
Electrochromic behaviors of EC Polyimides. **a** Absorption spectra of PI-1a. **b** Absorption spectra of PI-2a films. **c** Transmittance spectra of PI-1a. **d** Transmittance spectra of PI-2a films on ITO substrate. The insert photographs show the color changes of PI-1a and PI-2a films when the applied potential are changed from 0 to 1.3 V and 0 to 1.2 V, respectively

The EC behaviors of PI-1a and PI-2a films were investigated by spectroelectrochemistry experiments. In the neutral state (adjust the applied potential to 0 V), both polyimide films showed strong absorption peaks in the UV region, which were characterized as the π–π* transition of diamine moiety (shown in Fig. 3a, b)^19^. When the applied potential increased from 1.0 to 1.3 V, PI-1a film begin to oxidize and a board absorption peak at 796 nm continuously raised, exhibiting strong absorption in the entire visible region. Meanwhile, PI-2a film oxidized when the applied potential is between 0.9 and 1.2 V (shown in Fig. 3b) and a narrow absorption peak at 760 nm continuously increased. The oxidized PI-2a film showed weaker absorption intensity in the blue region than that in the green and red regions and almost no absorption in the near-IR region. The phenomena of broader absorption peak of oxidized PI-1a film (compared to PI-2a film) could be attributed to the longer conjugation length, which enhanced charge carrier delocalization compared to oxidized PI-2a film^19^.

The transmittance spectra and color changes of PI-1a and PI-2a films were also investigated. As shown in Fig. 3c, d, the transmittance spectra of PI-1a and PI-2a films in neutral states were both higher than 97% over most of the visible region, indicative of transparent and colorless appearance. When the applied potential on PI-1a film increased from 0 to 1.3 V, the transmittance spectra of PI-1a film dramatically decreased (see Fig. 3c) over the entire visible region and exhibited a black stage in the fully oxidized state. On the other hand, PI-2a film revealed a colorless-to-blue switching when the applied potential increased from 0 to 1.2 V (see Fig. 3d) due to its weak absorption in blue region (see Fig. 3b), viz. nearly half transmittance (~ 48%) at 470 nm still existed. Notably, the integrated contrast ratios (Δ%*T*_int_: integrated from 380 to 780 nm)^33^ of PI-1a and PI-2a were 91.4% and 81.2% (summarized in Table 1), respectively, which were much higher than that in literature^33^.

**Table 1:** Summary of contrast ratios, integrated contrast ratios and coloration efficiency

	PI-1a	PI-2a
Δ%*T* (*λ*_max_)	96.8% (at 798 nm)	96.2% (at 760 nm)
Δ%*T*_int_ (380–780 nm)	91.4%	81.2%
*η* [cm^2^ C^−1^] at *λ*_max_	99 (at 798 nm)	78 (at 760 nm)
*T*_400_ (%)	72	65

**Fig. 4:**
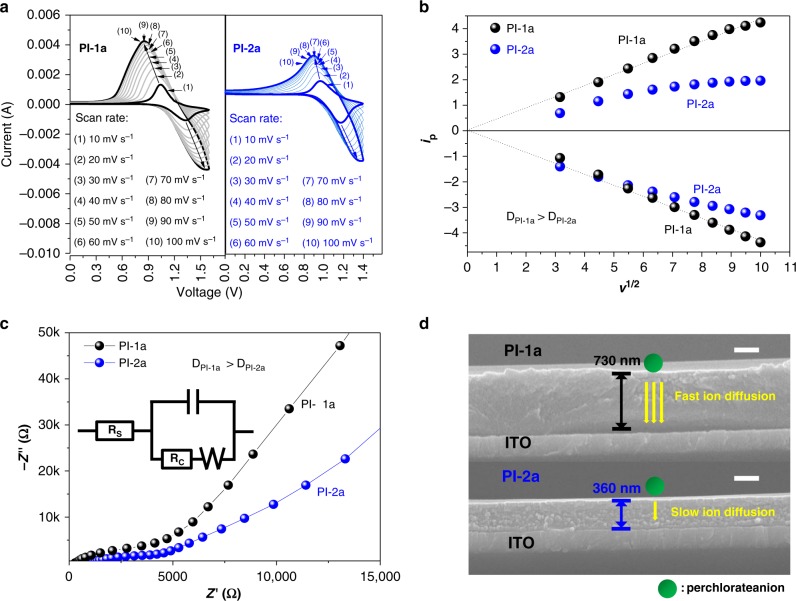
Electrochemical behaviors of EC Polyimides. **a** CVs of PI-1a and PI-2a films at different scan rates. **b** Plots of the peak current densities vs. (scan rates)^1/2^ of PI-1a and PI-2a films. **c** Nyquist diagrams of PI-1a and PI-2a films in 0.1 M TBAP/CH_3_CN solution. **d** Cross-sectional SEM images of PI-1a and PI-2a films. The scale bar is 200 nm

In order to investigate the charge transfer behaviors in PI-1a and PI-2a films, electrochemical response behaviors of PI-1a and PI-2a films were measured via scan rate alternation experiments and electrochemical impedance spectroscopy (EIS). First, the scan rate alternation experiments (from 10 to 100 mV) and the resulting peak currents vs. (scan rate)^1/2^ of PI-1a and PI-2a films were shown in Fig. 4a, b, respectively. In particular, the diffusion coefficient of counterion (${\mathrm{ClO}}_4^ -$) in both films could be analyzed according to the Randles–Sevcik equation (Eq. (1))^34^.

The current maximum value (*i*_p_) is proportional to the square root of scan rate (*v*^1/2^), with a slope of *kn*^3/2^ *AD*^1/2^*c*^34^. Therefore, *D* value (diffusion coefficient) of PI-1a (1.69 × 10^−7^ cm^2^ s^−1^) was higher than that of PI-2a films (5.41 × 10^−8^ cm^2^ s^−1^), indicating that the counterion (${\mathrm{ClO}}_4^ -$) diffusion in PI-1a film was faster than that in PI-2a film^19^. In addition, differential pulse voltammetry (DPV) of PI-1a and PI-2a films were performed and the results were shown in Supplementary Figure 12, which indicated that no significant electron coupling could be observed. Moreover, we also performed the electrochemical impedance spectroscopy (EIS) on PI-1a and PI-2a films, as shown in Fig. 4c. The higher slope of the PI-1a film at low frequency region indicated that the charge transfer in PI-1a electrochemical system was faster than that in PI-2a electrochemical system^19^.

### Fast EC behavior in PI-1a film

To understand the origin of the faster ionic transporting behavior and accelerated EC behavior of PI-1a film, SEM cross-section images of PI-1a and PI-2a films were measured (see Fig. 4d), and the thickness of PI-1a and PI-2a were 730 and 360 nm, respectively. Since we deposited the same amount of PI-1a and PI-2a on ITO within the same area (2 cm × 2 cm) and the molecular weight of single repeating unit (*M*_0_) of PI-1a (*M*_0_ = 1203) and PI-2a (*M*_0_ = 1223) were similar, the film thicknesses mainly depended on the polymer chain stacking property and were inversely proportional to the film density (Eq. (2))^35^.

The calculation methods of densities of PI-1a and PI-2a films were according to Methods and Supplementary Figure 13, which indicated the densities of two films (the homogeneously middle part) were 3.0 and 6.7 g cm^−3^, respectively. The density results demonstrated that PI-1a formed a more loosely stacking polymer film. The looser chain stacking of PI-1a could be attributed to the asymmetric (*N*-phenyl-*N*-phenyl) naphthalen-1-amine moiety^36^, which generated more disordered chain entanglement (Supplementary Figure 17) and free volumes^37^. In addition, the SAXS and XRD of PI-1a and PI-2a films were performed and the results were shown in Supplementary Figure 14, indicating that both of films were amorphous and there were no obviously close chain stacking^38–40^.

Generally, faster electrochromic response behaviors could be influenced by several factors: (1) higher conductivity of EC films^41^, (2) composition of electrolyte^42^, and (3) faster counterion diffusion^27,43^. When considering factor (1), polyimides are non-conjugated polymers and always used as high performance engineering plastic insulator (such as DUPONT™ KAPTON®). In addition, the large arc (*Z′*′(Ω): 0 ~ 2500) in the low frequency in EIS (Fig. 3c) demonstrated that both of the polyimide films exhibited very large resistence^44,45^, indicating that the difference in conductivity of PI-1a and PI-2a films could not be taken into consideration. That is, in principle, the two polyimide films are insulators having low conductivity. Factor (2) could also be omitted because PI-1a and PI-2a films are both tested in the same electrolyte (0.1 M TBAP/acetonitrile). In this contest, the faster electrochemical and electrochromic behavior might be considered as the faster counterion diffusion (factor (3)). Therefore, we further measured the different morphologies of PI-1a and PI-2a films by AFM (Supplementary Figure 15), which demonstrated that both films possessed similar roughness (below 1 nm). So the faster EC behaviors were not resulted from different morphologies. Based on the above discussion and results from SEM, CV, and EIS, we concluded that the faster counterion diffusion in PI-1a could be resulted from its lower density and higher free volume, providing more pathways for the penetration of counterion (${\mathrm{ClO}}_4^ -$)^19^.

**Fig. 5:**
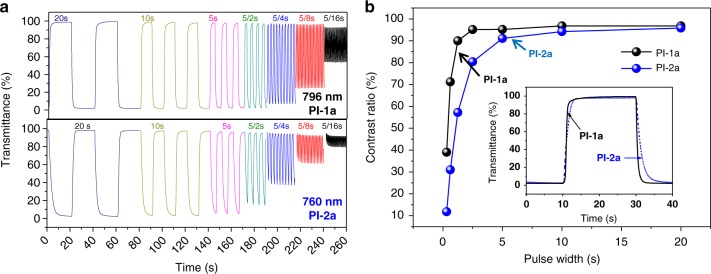
Transmittance changes of EC Polyimides. **a** Transmittance changes (760 nm, with decreasing pulse width from 20 to 5/16 s) of PI-1a and PI-2a films with applied potential between 0–1.3 and 0–1.2 V, respectively. **b** Contrast ratios vs. pulse width. Insert Figure shows the faster EC behavior of PI-1a

Further evidence of the faster charge transfer behavior in PI-1a film was demonstrated by the transmittance changes. As shown in Fig. 5a, continuous changes of pulse width were given to PI-1a and PI-2a films, their transmittance changes at 796 nm and 760 nm were monitored, respectively. The initial contrast ratio (with 20 s pulse width) of PI-1a and PI-2a films were both around 97%. The contrast ratio of PI-1a film decreased to 96%, 95%, 95%, 90%, 72%, and 39% when the applied pulse widths shortened to 10, 5, 5/2, 5/4, 5/8, and 5/16 s, respectively. On the contrary, the contrast ratio of PI-2a film decreased to 94, 91, 80, 57, 32, and 12% with the same applied pulse widths. Significantly, the higher contrast ratios of PI-1a film indicate that the PI-1a film has a more rapid counterion diffusion than PI-2a film, originated from the asymmetric pendant group ((*N*-phenyl-*N*-phenyl) naphthalen-1-amine) and resulting loose chain stacking. In addition, the contrast ratios *vs*. pulse widths of PI-1a and PI-2a films were further depicted in Fig. 5b for more direct comparison. Moreover, the switching (1.3 s) and bleaching (1.1 s) times of PI-1a are significantly slower than those of PI-2a films, plotted in Fig. 5b and summarized in Supplementary Table 2. The stability of transmittance change of PI-1a and PI-2a films were performed over 5000 cycles and shown in Supplementary Figure 16, indicating that the stability of PI-2a film is relatively higher than that of PI-1a.

### Colorimetry

**Fig. 6:**
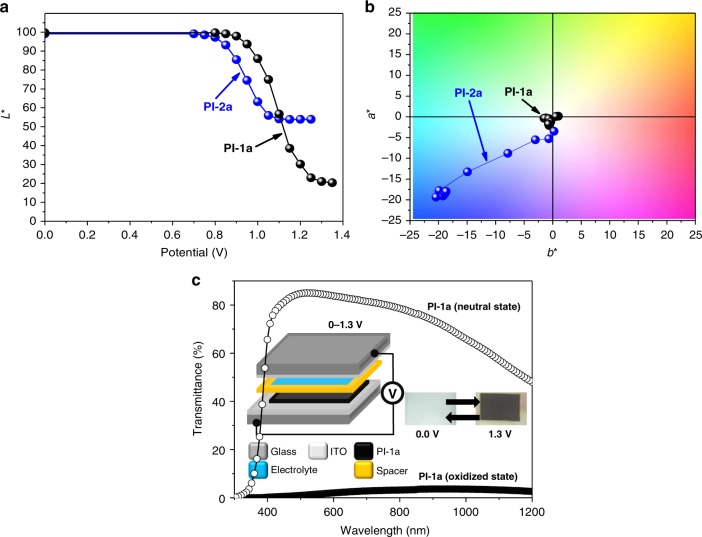
Color space results and EC device. **a** Lightness values and **b** a*b* color space results of PI-1a and PI-2a films. **c** Device transmittance of ITO/PI-1a/electrolyte/ITO

L*a*b* color space results for PI-1a and PI-2a films were presented in Fig. 6a, b. Both PI-1a and PI-2a films gave high L* values (more than 99%) in neutral states (see Fig. 6a), therefore, both films were highly transparent and colorless. In addition, oxidized PI-2a had a narrower, and unflat transmittance across the visible spectrum, which allowed unequal amounts of blue, green and red light to pass, thus gave a blue color. On the other hand, oxidized PI-1a film absorbed more blue light than PI-2a film, exhibiting a flatter transmittance in the visible light region, thus gave a black stage and a lower L* value. Upon electrochemical oxidation, the a*b* values of PI-1a film changed much smaller (from (0.2, 1.0) to (−1.7, −0.7)) than the those of PI-2a (from (−3.5, −0.2) to (−18, −18.7)), as shown in Fig. 6b and Supplementary Table 3). The L*a*b* color space results of PI-1a and PI-2a films indicated that the colorless-to-black and blue switching electrochromes could be achieved. In addition, Supplementary Figure 18 showed the photographs of PI-1a and PI-2a films in fully oxidized states, which were performed on flexible monolayer graphene electrodes (the fabrication method of graphene electrode^46,47^ was described in Methods), which exhibited good coloration properties, indicative of their versatility on different electrodes.

## Discussion

The two polyimides exhibited much higher contrast ratios than the previously reported ECPs, including conjugated polymers, polyimides and polynorbornenes^18,20,48,49^. Therefore, the difference between color points, denoted as $\Delta E_{ab}^ \ast$, was calculated for determining the significance of color change observed by human eyes according to Eq. (3)^33^. The values of $\Delta E_{ab}^ \ast$ for PI-1a and PI-2a between neutral states and oxidized states were calculated to be 79 and 49, respectively, indicating the more remarkable color change of PI-1a. The coloration efficiency of PI-1a film was also calculated to be 99 cm^2^ C^−1^ by using Eq. (4), which is higher than that of PI-2a film (78 cm^2^ C^−1^) due to the faster charge transfer in electrochemical system^19^. To further elaborate the high Δ%*T*_int_ (integrated contrast ratio of transmittance changes) of PI-1a film, EC device of PI-1a was fabricated with device architecture of ITO/PI-1a/electrolyte/ITO^50^. The transmittance spectra of the device when PI-1a was in neutral and oxidized states are shown in Fig. 6c. The transmittance spectra revealed an ultrahigh integrated contrast ratio (Δ%*T*_int_) up to 80%.

In summary, colorless-to-colorful EC polyimides with twisted and adjusted conjugated structures were prepared via architectural design. The L*a*b* color space results of EC polyimide films indicated that the colorless-to-colorful switching electrochromes were achieved, which was very unique in the field of developing EC color display and optical areas. In particular, PI-1a and PI-2a films were colorless in visible region. On the other hand, PI-1a and PI-2a films exhibited black and blue colors in fully oxidized state, respectively. In addition, we also showed that the charge transfer behaviors were strongly depending on the chemical structures. Moreover, we proved that a fast counterion diffusion in PI-1a film due to the asymmetric structure, which resulted in loosely chain stacking morphology and fast EC responses. Therefore, the facile strategy in developing colorless-to-colorful EC polyimides are very attractive to the field of EC display and optical areas.

## Methods

### Basic characterization

The NMR measurements were carried out on a Bruker DRX-500 at 600 MHz for ^1^H NMR and 150 MHz for ^13^C NMR spectra. Absorption and transmittance spectra were performed on a JASCO-V-670 spectrophotometer. The background of ITO or glass (where the thin film were deposited) was subtracted when we tested the absorption and transmittance spectra of polymer films. Photoluminescence excitation spectra of polymer films were recorded on a HORIBA Jobin Yvon FluoroMax-3 spectrofluorometer. Cyclic voltammetry (CV) was conducted with CHI model 619A. ITO and a platinum wire were used as a working electrode and an auxiliary electrode, respectively. The CV and EC experiments were performed in a solution of 0.1 M tetrabutylammonium perchlorate (TBAP)/acetonitrile (CH_3_CN) against a Ag/Ag^+^ reference electrode. High resolution field-emission scanning electron microscope (SEM) images of polymer films were conducted on JEOL JSM-6500F. EIS measurements of polymer films were performed on BCS-815 with the frequency range of 10 mHz–10 kHz. DPV of PI-1a and PI-2a films were performed on Autolab PGSTATE302 MBA. XRD was performed on D2 PHASER (BRUKER). SAXS was performed on an in-house Bruker Nanostar SAXS instrument. AFM was performed on Dimension Icon (BRUKER).

### Film preparation

All the thin films described in this study were prepared in the following procedure: 10 mg polyimides were dissolved in 1 ml NMP; the solution was stirred at 85 °C for 1 h. Hundred microliters of the above solution was drop-cast on a 2 × 2 cm^2^ ITO-coated substrate and annealed at 85 °C for 1 h and 110 °C for another 1 h in sequence. All the procedures are carried out under ambient conditions. The films described in Fig. 1c, e were prepared using a more concentrated solution (0.1 g ml^−3^) and the films for refractive index were deposited on silicon wafers. The thicknesses of PI-1a and PI-2a films are 730 and 360 nm for the measurement of EC experiments, SEM and AFM, respectively.

The preparation of CVD Graphene/PET films were described as follows. On a 25 mm copper foil (Alfa Aesar), we used CVD method to grow large-area few-layered graphene. Before growth, we used plasma to remove the oxide layer of copper. At graphene growth temperature (1000 °C), we introduced a flow of 15 sccm H_2_ and 65 sccm CH_4_. After CVD growth of graphene, we used a transfer process with PMMA (15k, 4.6 wt%) coating to transfer graphene electrode on to PET substrate. We use Ferric Nitride (0.05 mg ml^−1^) to etch away the copper foil substrate and the graphene/PMMA thin film was floating on etchant solution. We washed the PMMA film by D.I. water and transferred it onto PET substrates (with a sheet resistance of ∼1k–2k Ω/sq and a transmittance of 97.5% at 550 nm). We also used absorption spectrum to confirm that less than three layers of graphene were formed on PET substrate, because the graphene/PET showed a transmittance of 94–97% at 550 nm and a monolayer graphene absorbed ∼2.3% transmittance at 550 nm.

### EC device fabrication

For the preparation of gel electrolyte of EC device, we mixed polyethylene glycol (15 kDa) and LiClO_4_ (with a molar ratio of 8:1) and dissolved them in 25% w/v acetonitrile. The acetonitrile solvent was stirred and evaporated at 25 °C before the polyethylene glycol precipitated. We placed a adhesive tape on the ITO (2 cm × 2 cm) to form a frame and dropped the gel electrolyte within the adhesive tape frame. Then we placed the polyimide coated ITO glass face-down on the gel-coated slide, and pressed the two electrodes together using clips.

### Diffusion coefficient

We analyzed the diffusion coefficient of counterion (${\mathrm{ClO}}_4^ -$) in both PI-1a and PI-2a films according to the Randles–Sevcik equation, described as follows:

1 $$i_{\mathrm{p}} = kn^{\frac{3}{2}}AD^{\frac{1}{2}}cv^{\frac{1}{2}}$$

The current maximum value (*i*_p_) of polymer film was proportional to the square root of scan rate (*v*^1/2^), the slope is *kn*^3/2^*AD*^1/2^*c*.

### Film density

The film thicknesses mainly depended on the polymer chain stacking property and were inversely proportional to the film density:

2 $${\mathrm{Density}}_{\mathrm{polyimide}} = \frac{{\mathrm{Weight}}_{\mathrm{Film}}}{{\mathrm{Volume}}_{{\mathrm{Film}}}} = \frac{{\mathrm{Weight}}_{\mathrm{Film}}}{{\mathrm{Area}}_{\mathrm{Film}} \times {\mathrm{Thichkness}}_{\mathrm{Film}}}$$

The calculation of polymer densities of PI-1a and PI-2a films were firstly cut off the edge of five pieces of polymer films (for each piece polymer film is one milligram) because the edge is thicker than the middle part (see Supplementary Figure 9), the resulting edge of five PI-1a and PI-2a films was weight to be 2.2 and 1.9 mg, respectively. The densities of PI-1a and PI-2a films were calculated according to Eq. (2):

$${\mathrm{Density}}_{{\mathrm{PI}} - 1{\mathrm{a}}} = \frac{{\mathrm{total}} \hskip3pt {\mathrm{Weight}}_{{\mathrm{PI}} - 1{\mathrm{a}}}({\mathrm{five}} \hskip3pt {\mathrm{pieces}})}{{\mathrm{total}} \hskip3pt {\mathrm{Volume}}_{{\mathrm{PI}} - 1{\mathrm{a}}}({\mathrm{five}} \hskip3pt {\mathrm{pieces}})} = \frac{5.0 \hskip3pt {\mathrm{mg}} - 2.2 \hskip3pt {\mathrm{mg}}}{9.344 \times 10^{ - 4} \hskip3pt {\mathrm{cm}}^3} = 3.0 \hskip3pt {\mathrm{g}} \hskip3pt {\mathrm{cm}}^{ - 3}$$

$${\mathrm{Density}}_{{\mathrm{PI}} - 2{\mathrm{a}}} = \frac{{\mathrm{total}} \hskip3pt {\mathrm{Weight}}_{{\mathrm{PI}} - 2{\mathrm{a}}}({\mathrm{five}} \hskip3pt {\mathrm{pieces}})}{{\mathrm{total}} \hskip3pt {\mathrm{Volume}}_{{\mathrm{PI}} - 2{\mathrm{a}}}({\mathrm{five}} \hskip3pt {\mathrm{pieces}})} = \frac{5.0 \hskip3pt {\mathrm{mg}} - 1.9 \hskip3pt {\mathrm{mg}}}{4.608 \times 10^{ - 4} \hskip3pt {\mathrm{cm}}^3} = 6.7 \hskip3pt {\mathrm{g}} \hskip3pt {\mathrm{cm}}^{ - 3}$$

### Difference between color points

The difference between color points, denoted as $\Delta E_{ab}^ \ast$, was calculated for determining the significance of color change observed by human eyes, as following equation:

3 $$\Delta E_{ab}^ \ast = \sqrt {\left( {\Delta L^ \ast } \right)^2 + \left( {\Delta a^ \ast } \right)^2 + \left( {\Delta b^ \ast } \right)^2}$$

### Coloration efficiency

The coloration efficiency of polyimide films were calculated according to the following equation:

4 $${\mathrm{Coloration}} \hskip3pt {\mathrm{Efficiency}} = \frac{1}{Q} {\mathrm{log}} \frac{\tau_{b}}{\tau_{c}}$$

where *Q* is the insertion charges, and the *T*_b_ and *T*_c_ are transmittances of colored and bleached states of EC films.

## Supplementary information

Supplementary Information

## Data Availability

The data that support the findings of this study are available from the authors on reasonable request.
