# Supplementary material for: Colorless-to-colorful switching electrochromic polyimides with very high contrast ratio
Source: Nat Commun. 2019 Mar 18;10:1239. doi: 10.1038/s41467-019-09054-8 (PMC6423275; doi:10.1038/s41467-019-09054-8)
Supplement: Supplementary file 1 — Supplementary Information [file 41467_2019_9054_MOESM1_ESM.pdf]

Supplementary Information

Colorless-to-colorful switching electrochromic polyimides with very high contrast ratio

Qiang Zhang [1,†], Chou-Yi Tsai [1,†], Lain-Jong Li [2] and Der-Jang Liaw [1,*]

Affiliations:

[1] Department of Chemical Engineering, National Taiwan University of Science and Technology, 10607 Taipei, Taiwan

[2] School of Materials Science and Engineering, University of New South Wales, NSW 2052, Australia

Correspondence and requests for materials should be addressed to D.-J. L. (email: liawdj@gmail.com; liawdj@mail.ntust.edu.tw)

† These authors contributed equally to this work

Supplementary Methods

Materials

Acetic acid glacial (Merck), Zn powder (Merck), sulfonic acid (Alpha, 98%), acetic anhydride (Merck), pyridine (Merck), nitrobenzene (TCI, 98%), *p*-bromoanisole (Aldrich, 99%), 1,1'-bis(diphenylphosphino)ferrocene (DPPF; Acros, 98%), 2-isopropoxy-4,4,5,5-tetramethyl-1,3,2-dioxaborolane (Aldrich, 98%), bromobenzene and bromonaphthalene (Acros), 9-bromoanthracene (Aldrich, 94%), *n*-butyllithium (Albemarle, 2.5 M in hexanes), sodium *tert*-butoxide (Acros, 98%), tetrakis(triphenylphosphine)palladium(0) ($Pd(PPh_3)_4$) (Acros, 99%), bis(dibenzylideneacetone)palladium(0) ($Pd(dba)_2$) (Acros, 96%) and anhydrous potassium carbonate (Fisher Scientific, ≥99%) were purchased and used as received from commercial sources. Tetrahydrofuran and toluene were purchased from Merck, and were dried and distilled over sodium metal under inert nitrogen atmosphere. *N*-Methyl-2-pyrrolidone was purchased from Merck and dried with calcium hydride (Acros) over night, then distilled and kept under dried nitrogen atmosphere. Acetic anhydride and pyridine were purchased from Merck. 4,4'-(Hexafluoroisopropylidene)diphthalic anhydride (6FDA) was purchased from Sigma-Aldrich and purified through sublimation.

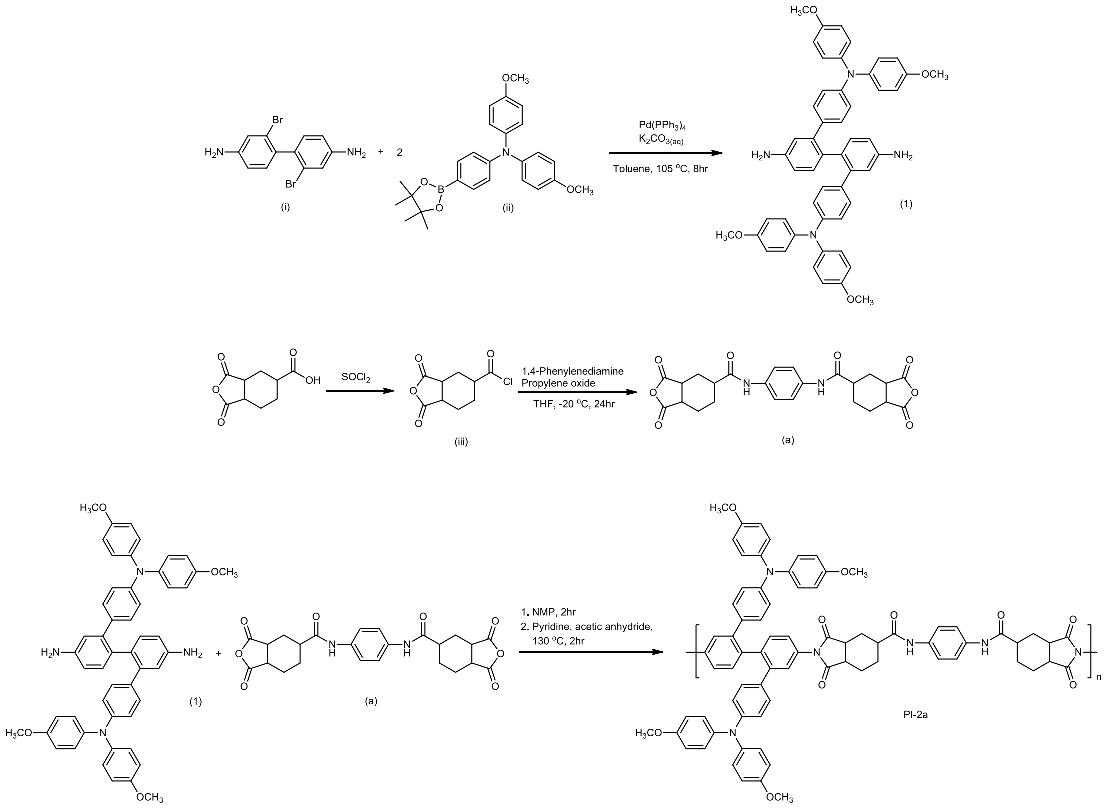

Supplementary Figure 1. Synthetic route of PI-2a.

Synthesis of monomer (1)

Monomer (1) was prepared *via* Suzuki-Miyaura coupling reaction. A three-necked flask thoroughly evacuated and filled with nitrogen gas was charged with 4-methoxy-N-(4-methoxyphenyl)-N-(4-(4,4,5,5-tetramethyl-1,3,2-dioxaborolan-2-yl)phenyl)aniline (2) (1.9 g, 4.4 mmole), 2,2'-dibromo-[1,1'-biphenyl]-4,4'-diamine (0.7 g, 2 mmole),[1-3] and 10 mL of degassed toluene. After monomers dissolution, tetrakis(triphenylphosphine) palladium(0) ($Pd(PPh_3)_4$, 92 mg, 0.08 mmole) and sodium carbonate aqueous solution (10 mL, 3M) were added to the flask. The mixture was stirred at 105 °C for 8 h under inert nitrogen atmosphere. After reaction completion, the reaction mixture was gradually cooled to room temperature. The organic layer was collected, washed with water and toluene was evaporated. The resultant residue was purified by column chromatography (silica gel, dichloromethane) to obtain Monomer (1) as a white solid (71% yield, 0.33 g). ^1H NMR (600 MHz, $CDCl_3$), δ_H (ppm): 7.16-7.17 (d, 2H, H_{14}), 6.97-6.99 (m, 8H, H_3), 6.72-6.75 (m, 8H, H_4), 6.64-6.66 (m, 6H, H_8 and H_{13}), 6.54-6.56 (m, 6H, H_7 and H_{11}), 3.76 (s, 12H, H_1), 3.61 (s, 4H, amine proton). ^{13}C NMR (150 MHz, $CDCl_3$), δ_C (ppm): 55.3 (C_1), 113.8 (C_{13}), 114.5 (C_4), 116.2 (C_{11}), 120.8 (C_8), 125.8 (C_3), 129.4 (C_7), 130.9 (C_{15}), 132.6 (C_{14}), 134.4 (C_9), 141.2 (C_5 and C_{10}), 145.1 (C_{12}),

146.5 (C_6), 155.3 (C_2).

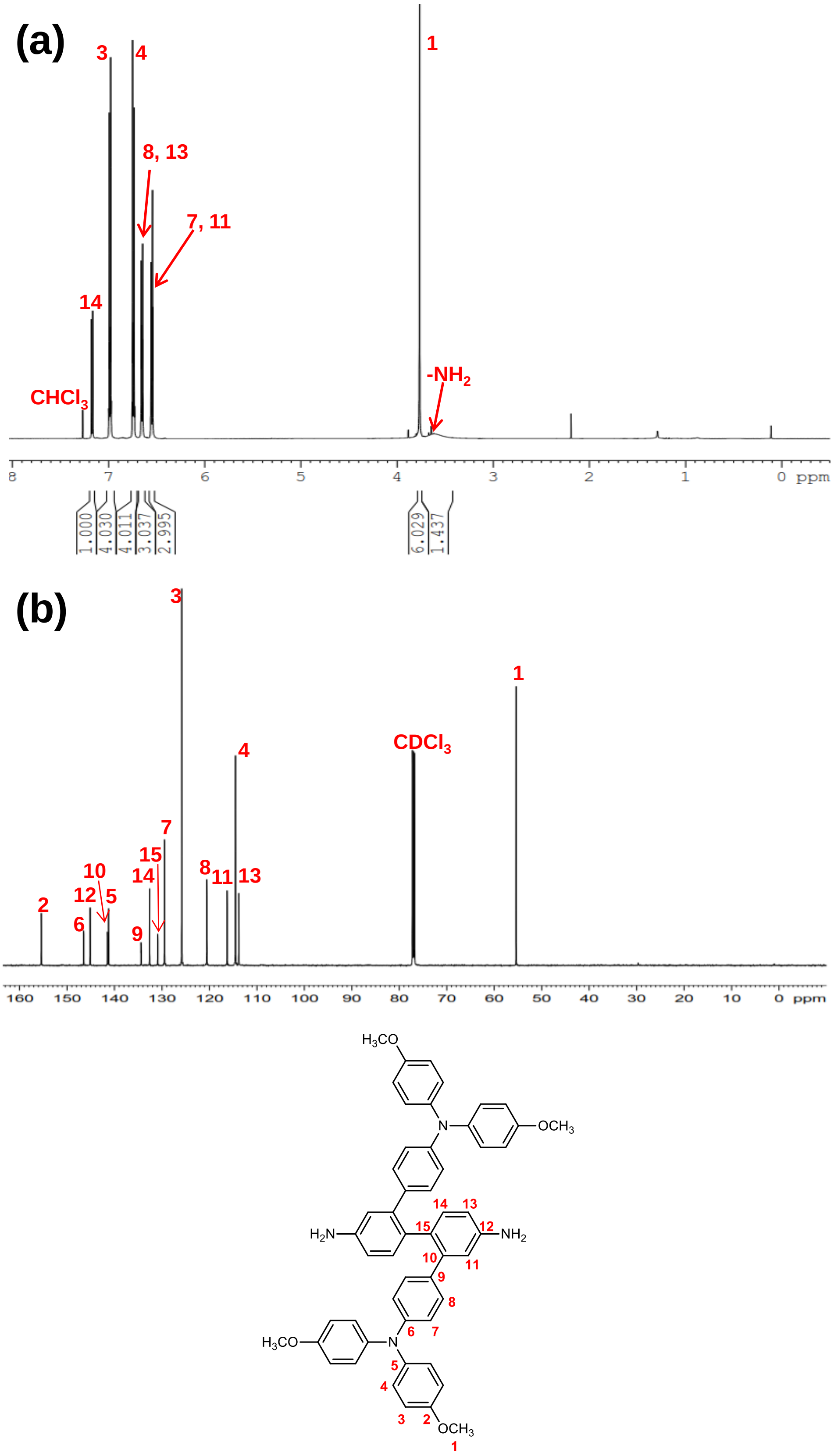

Supplementary Figure 2. NMR spectra of monomer (1). **(a)** ^{1}H NMR and **(b)** ^{13}C NMR.

Synthesis of monomer (iii)

1,2,4-Cyclohexanetricarboxylic anhydride (H-TMAn, 19.8 g, 100 mmol) was chlorinated with thionyl chloride (40 ml) by refluxing at 80 ^{o}C for 2 h. The excess of thionyl chloride was azeotropically removed by adding toluene under a reduced pressure. The resultant residue was purified by glass oven at 120 ^{o}C in vacuum to obtain monomer (iii) as a transparent liquid (60% yield, 13 g). ^{1}H NMR (600 MHz, $CDCl_3$), δ_H (ppm): 3.40-3.43 (m, H_{2T}), 3.26-3.29 (m, H_{2C}), 3.12-3.19 (m, H_1), 2.85-2.90 (m, H_{4T}), 2.76-2.81 (m, H_{4C}), 2.52-2.56 (m, $H_{6C\alpha}$), 2.38-2.42 (m, $H_{3T\alpha}$), 2.32-2.36 (m, $H_{3C\alpha}$), 2.08-2.20 (m, $H_{5C\alpha}$, $H_{5C\beta}$, and $H_{6T\alpha}$), 2.03-2.07 (m, $H_{3T\beta}$), 1.78-1.83 (m, $H_{3C\beta}$), 1.63-1.74 (m, $H_{5T\beta}$, $H_{6T\beta}$, and $H_{6C\beta}$), 1.52-1.59 (m, $H_{5C\beta}$). ^{13}C NMR (150 MHz, $CDCl_3$), δ_C (ppm): 20.4 (C_{3T}), 22.9 (C_{6T}), 23.9 (C_{3T}), 24.6 (C_{5T}), 24.7 (C_{5C}), 27.1 (C_{6C}), 39.1 (C_{2T}), 39.2 (C_{1C}), 39.3 (C_{1T}), 39.5 (C_{2C}), 49.4 (C_{4T}), 50.4 (C_{4C}), 171.15 (C_{7C}), 171.6 (C_{7T}), 175.4 (C_8), (C_{9C}), 175.4 (C_{9T}).

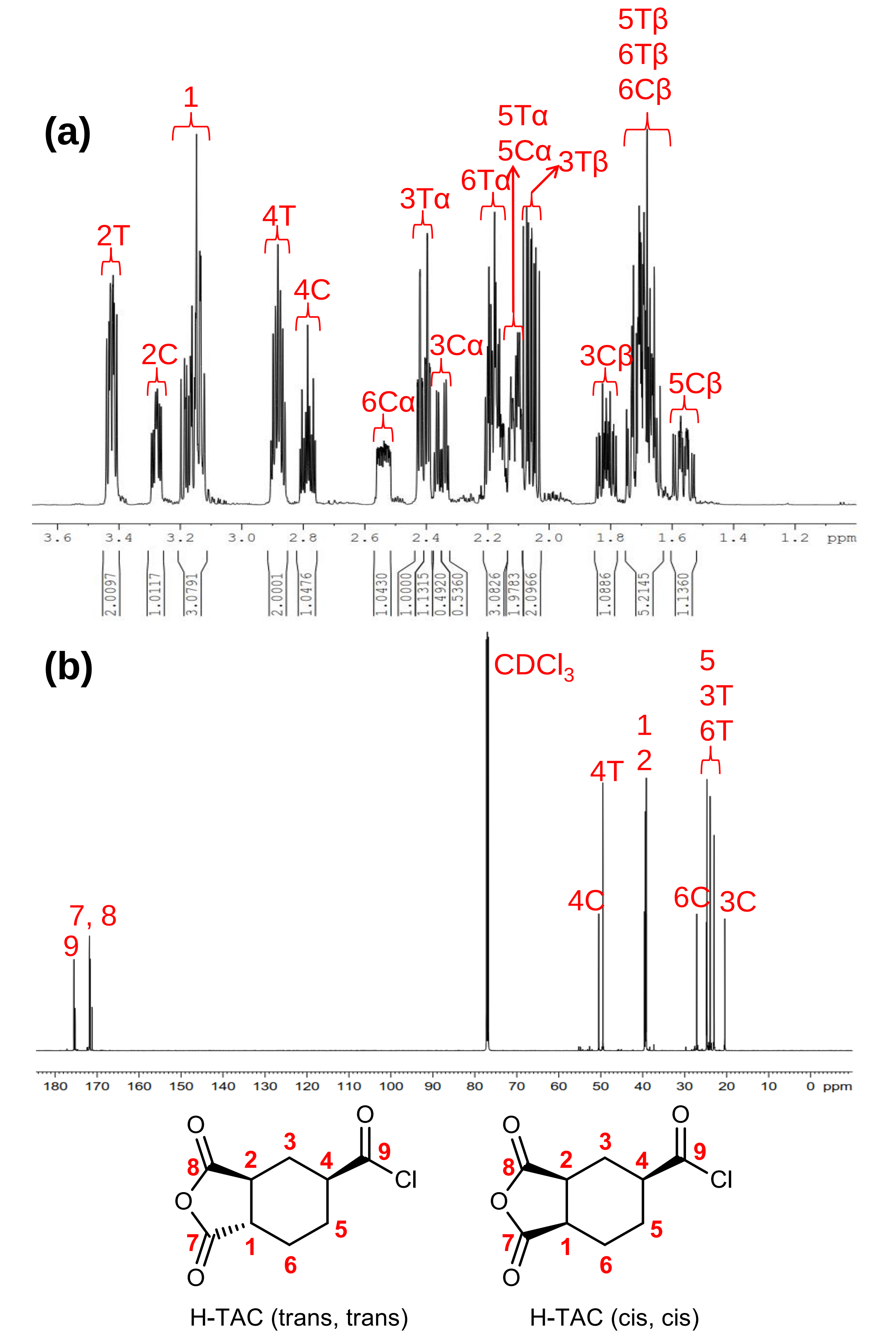

Supplementary Figure 3. NMR spectra of monomer (iii). **(a)** ^{1}H NMR and **(b)** ^{13}C NMR.

Synthesis of Monomer (a, HTA-PPD)

Monomer (iii) (10.8 g, 50 mmole) was dissolved in 50 mL anhydrous tetrahydrofuran (THF) in a septum-sealed flask. In a separate sealed flask, *p*-phenylenediamine (2.7 g, 25 mmole) was dissolved in 50 mL anhydrous THF in the presence of 3.5 mL propylene oxide (50 mmole) as an HCl acceptor. The diamine solution was slowly added to the HTAC solution kept at -20 ℃ using a syringe with continuous magnetic stirring for 2 h, and the reaction mixture was additionally stirred at room temperature for 24 h. The precipitated product was filtered. The product was recrystallized twice from anhydrous mixer solvent (THF/EA = 4/1 v/v), and vacuum-dried again at 140 ℃ for 12 h to obtain monomer (a) as a white solid (30% yield, 3.5 g). ^{1}H NMR (600 MHz, DMSO-d_6), δ_H (ppm): 9.82-9.89 (m, 2H, H_{12}), 7.47-7.51 (m, 4H, H_{11}), 3.68-3.71 (m, 1H, H_{2T}), 3.52-3.55 (m, 1H, H_{2C}), 3.23-3.27 (m, 2H, H_1), 2.35-2.41 (m, 2H, H_4), 2.05-2.13 (m, 4H, $H_{3T\alpha}$, $H_{3C\alpha}$, $H_{6T\alpha}$ and $H_{6C\alpha}$), 1.85-1.90 (m, 1H, $H_{3T\beta}$), 1.75-1.80 (m, 3H, $H_{3C\beta}$, $H_{5T\alpha}$ and $H_{5C\alpha}$), 1.54-1.61 (m, $H_{6C\beta}$), 1.42-1.53 (m, $H_{5T\beta}$ and $H_{6T\beta}$), 1.36-1.40 (m, $H_{5C\beta}$). ^{13}C NMR (150 MHz, DMSO-d_6), δ_C (ppm): 20.1 (C_{3T}), 23.4 (C_{3T}), 23.7 (C_{6T}), 25.0 (C_5), 27.1 (C_{6C}), 38.8 (C_2), 39.0 (C_1), 39.7 (C_4), 119.6 (C_{11}), 134.5 (C_{10}), 172.4 (C_9), 173.3, 173.6, 174.1, 174.2 (C_7 and C_8).

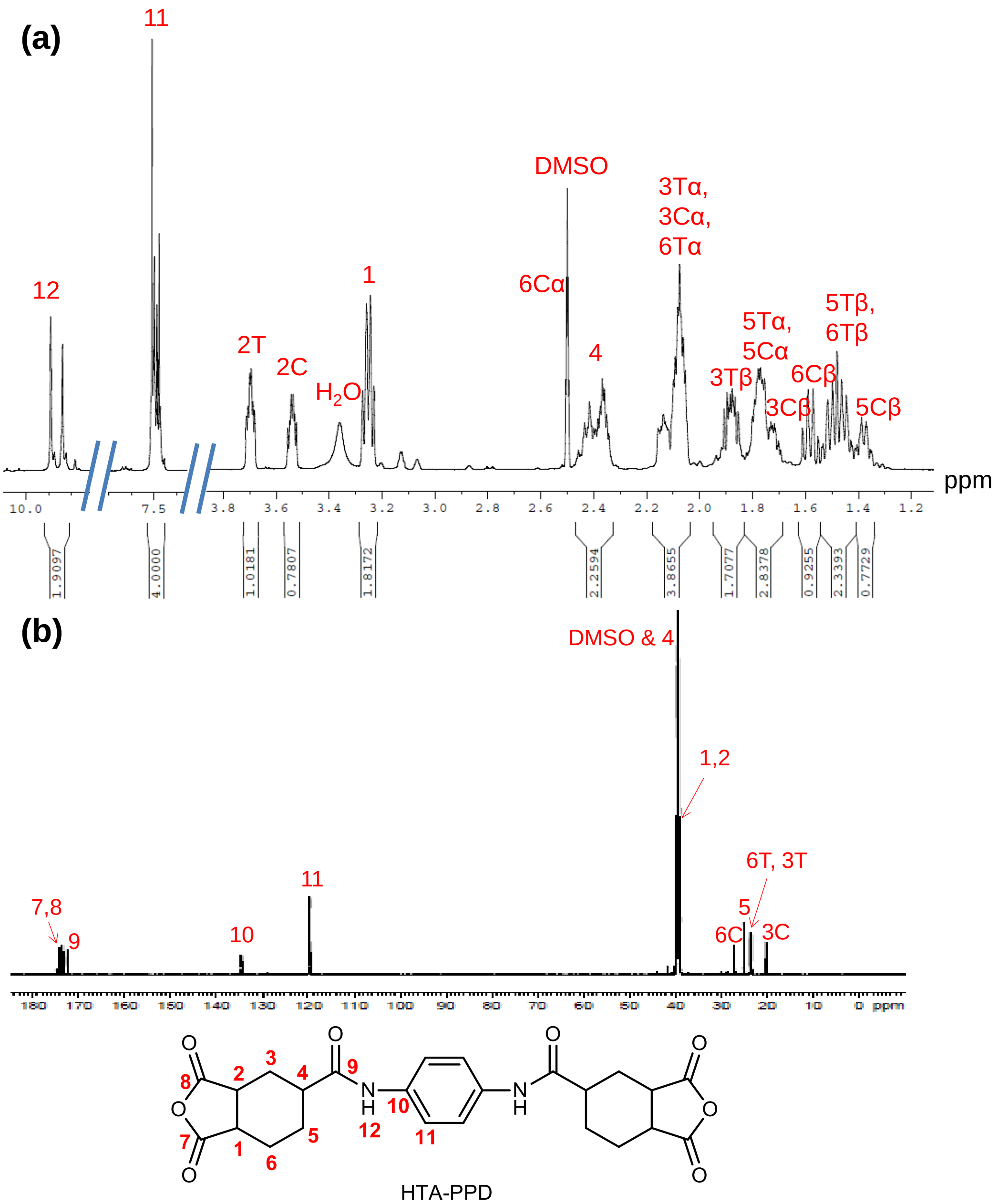

HTA-PPD

T = Trans, trans-1,2,4-cyclohexanetricarboxylic acid-1,2-anhydride

C = cis, cis-1,2,4-cyclohexanetricarboxylic acid-1,2-anhydride

Supplementary Figure 4. NMR spectra of monomer (a). **(a)** ^{1}H NMR and **(b)** ^{13}C NMR.

Synthesis of compound PI-2a

To a stirred solution of 0.40 g (0.5 mmole) of Monomer (1) in 5 mL of *N*-methyl-2-pyrrolidinone (NMP), 0.23 g (0.5 mmole) of Monomer (a) was gradually added. The mixture was stirred at ambient temperature for 8 h to form the poly(amic acid). Chemical cyclodehydration was carried out by addition of 1 mL of acetic anhydride and 0.5 mL of pyridine into the above-mentioned poly(amic acid) solution with stirring at room temperature for 1 h, and then heating at 130 ℃ for 2 h. Then the polymer solution was poured into methanol. The precipitate was filtered, washed with methanol, and dried at 100 ℃ under vacuum to obtain PI-2a (0.5 g, 98%) as white solid. ^{1}H NMR (600 MHz, DMSO-d_6), δ_H (ppm): 9.84 (s, 2H, H_{27}), 7.49-7.55. (m, 6H, H_{14} and H_{26}), 7.34 (s, 2H, H_{13}), 7.16-7.19 (d, 2H, H_{11}), 6.90–6.91 (d, 8H, H_3), 6.79 (s, 8H, H_4), 6.48–6.53 (d, 8H, H_7 and H_8), 3.68 (s, 12H, H_1), 3.28-3.41 (m, 2H, H_{17}), 3.09 (s, 2H, H_{16}), 2.42 (m, 2H, H_{19}), 1.44–2.18 (m, 12H, H_{18}, H_{20} and H_{21}). ^{13}C NMR (150 MHz, DMSO-d_6), δ_C (ppm): 20.3 (C_{18C}), 23.8, 23.9 (C_{18T} and C_{21T}), 24.8, 24.9 (C_{20T} and C_{20T}), 28.3 (C_{21C}), 39.0 (C_{16}, C_{17} and C_{19}), 55.0 (C_1), 114.7 (C_4), 119.5 (C_8 and C_{26}), 125.0 (C_{13}), 125.8 (C_3), 127.3 (C_{11}), 129.2 (C_7), 131.7 (C_{10} and C_{14}), 134.5 (C_{25}), 138.3 (C_{12}), 140.0 (C_5), 140.1, 140.4 (C_9 and C_{15}), 146.7 (C_6), 155.3 (C_2), 172.85, 173.01 (C_{24}), 177.8, 178.1, 178.5, (C_{22} and C_{23}). IR (KBr): 3320 cm^{-1} (amide N-H stretch), 3037 cm^{-1} (sp^2 C-H stretch), 2935 cm^{-1} & 2835 cm^{-1} (sp^3 C-H stretch), 1668 cm^{-1} (amide C=O stretch), 1604 & 1477 cm^{-1} (C=C aromatic stretch), 827 cm^{-1} (aromatic C-H out of plane). PI-1a exhibited a T_g of 260 °C and a $T_{d10\%}$ of 438 °C under nitrogen flow. PI-1a showed a inherent viscosity of 1.91, measured in NMP at a concentration of 0.5 g dL^{-1} at 30 °C.

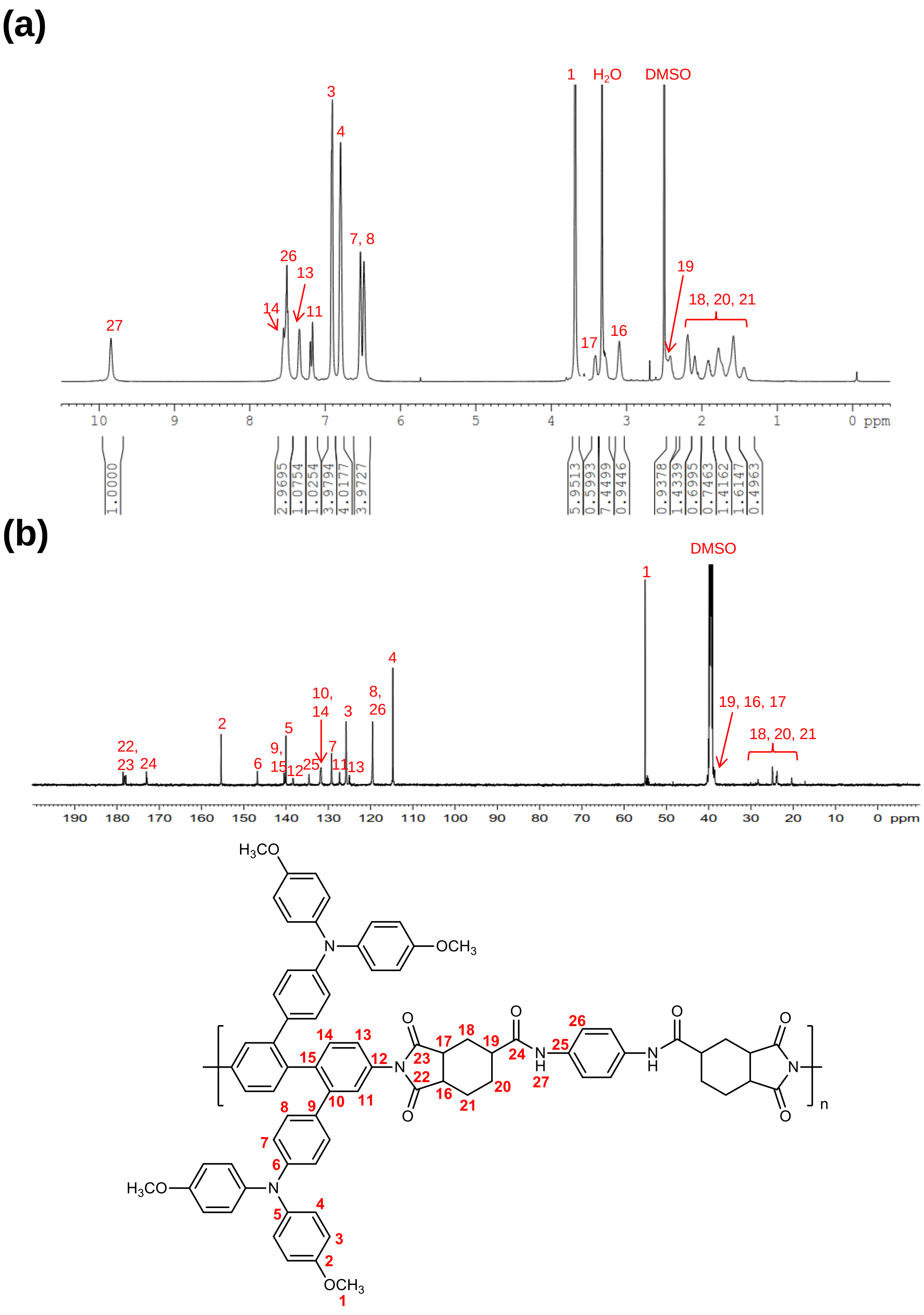

Supplementary Figure 5. NMR spectra of PI-2a. **(a)** ^{1}H NMR and **(b)** ^{13}C NMR.

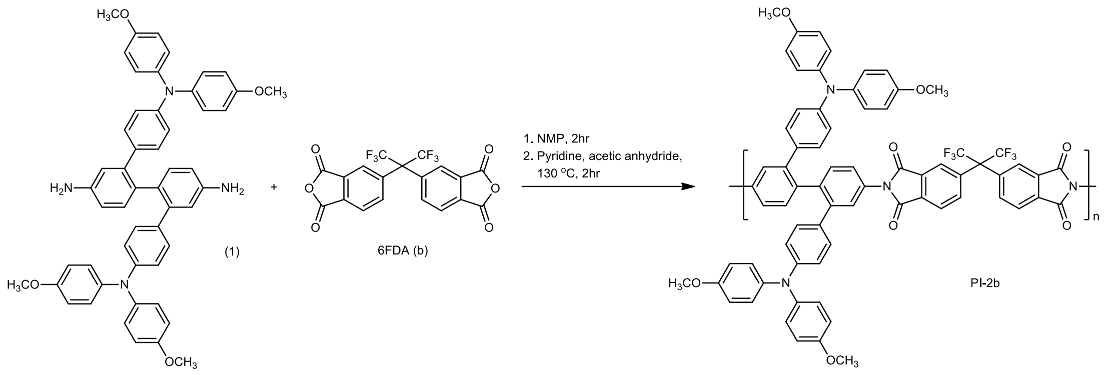

Supplementary Figure 6. Synthetic route of PI-2b.

Synthesis of compound PI-2b

To a stirred solution of 0.40 g (0.5 mmole) of Monomer (1) in 5 mL of *N*-methyl-2-pyrrolidinone (NMP), 0.22 g (0.5 mmole) of 4,4'-(hexafluoroisopropylidene)diphthalic anhydride (6FDA, (b)) was gradually added. The mixture was stirred at ambient temperature for 8 h to form the poly(amic acid). Chemical cyclodehydration was carried out by addition of 1 mL of acetic anhydride and 0.5 mL of pyridine into the above-mentioned poly(amic acid) solution with stirring at room temperature for 1 h, and then heating at 130 °C for 2 h. Then the polymer solution was poured into methanol. The precipitate was filtered, washed with methanol, and dried at 100 °C under vacuum to obtain PI-2b (0.58 g, 98%) as yellow-brown solid. ^{1}H NMR (600 MHz, THF-d_8), δ_H (ppm): 8.11 (d, 2H, H_{21}), 8.10 (d, 2H, H_{20}), 7.99 (d, 2H, H_{18}), 7.60-7.62 (t, 2H, H_{14}), 7.53-7.54 (d, 2H, H_{13}), 7.43 (s, 2H, H_{11}), 6.93-6.94 (d, 8H, H_3), 6.74-6.75 (d, 8H, H_4), 6.58-6.64 (m, 8H, H_7 and H_8), 3.63 (s, 12H, H_1). ^{13}C NMR (150 MHz, THF-d_8), δ_C (ppm): 55.5 (C_1), 67.6 (C_{24}), 115.4 (C_4), 121.1 (C_8), 124.6 (C_{18}), 125.2 (C_{25}), 125.2 (C_{21}), 126.9 (C_3,C_{13}), 127.9 (C_{11}), 130.6 (C_7), 132.7 (C_9, C_{10}), 133.4 (C_{14}), 134.0 (C_{16}), 134.4 (C_{17}), 136.7 (C_{20}), 139.3 (C_{19}), 140.0 (C_{12}), 141.8 (C_5), 142.4 (C_{15}), 148.5 (C_6), 157.0 (C_2), 166.6 (C_{22}, C_{23}).

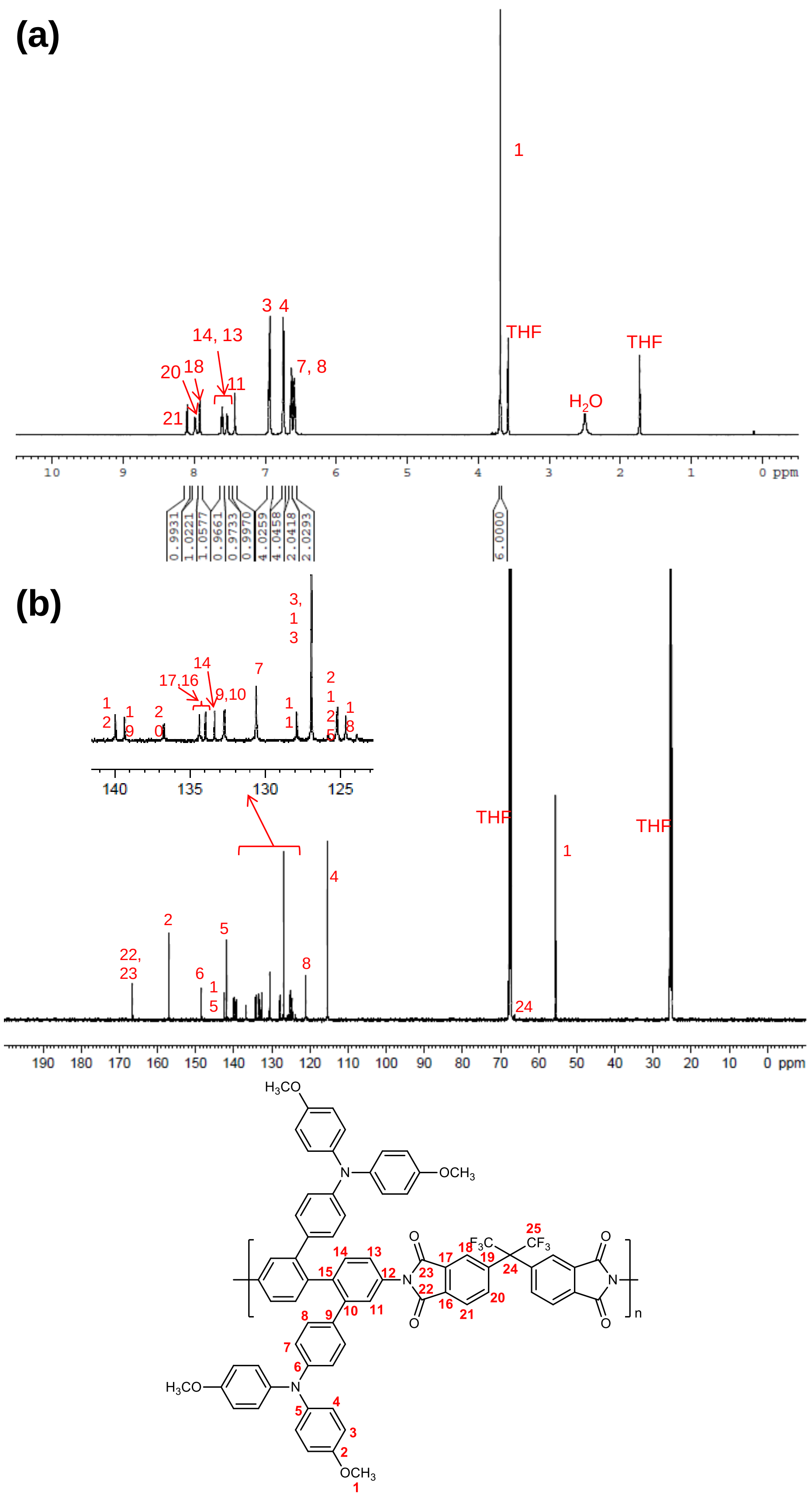

Supplementary Figure 7. NMR spectra of PI-2b. **(a)** ^{1}H NMR and **(b)** ^{13}C NMR.

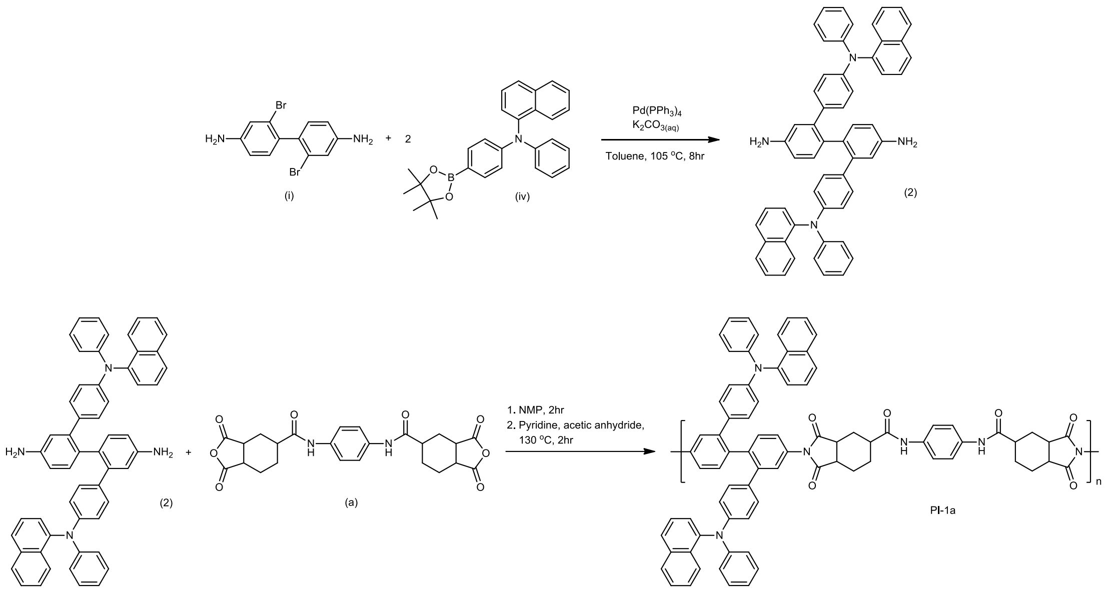

Supplementary Figure 8. Synthetic route of PI-1a.

Synthesis of monomer (2)

Monomer (2) was synthesized through Suzuki-Miyaura coupling reaction, viz. coupling reaction of 2,2'-dibromo-[1,1'-biphenyl]-4,4'-diamine (Monomer (i)) and *N*-phenyl-*N*-(4-(4,4,5,5-tetramethyl-1,3,2-dioxaborolan-2-yl)phenyl)naphthalen-1-amine (Monomer (iv)).[4] A three-necked flask thoroughly evacuated and filled with nitrogen gas was charged with Monomer (i) (0.68 g, 2 mmole), Monomer (iv) (1.69 g, 4 mmole) and 7 mL of degassed toluene. After monomers dissolution, tetrakis(triphenylphosphine)palladium(0) $Pd(PPh_3)_4$ (92 mg, 0.08 mmole) and sodium carbonate solution (7 mL, 3M) were added to the flask. The mixture was stirred at 105 °C for 8 h under inert nitrogen atmosphere. After reaction completion, the reaction mixture was gradually cooled to room temperature. The organic layer was collected, washed with water and toluene was evaporated. The resultant residue was purified by column chromatography (silica gel, dichloromethane) to obtain Monomer (2) as a white solid (0.40 g, 26%). ^{1}H NMR (600 MHz, $CDCl_3$), δ_H (ppm): 7.87 (d, 2H, H_4), 7.78-7.79 (d, 2H, H_9), 7.66-7.67 (d, 2H, H_6), 7.30-7.40 (m, 6H, H_3, H_7 and H_8), 7.22 (d, 2H, H_2), 7.15-7.16 (d, 2H, H_{14}), 7.06-7.09 (m, 4H, H_{13}), 6.89 (d, 2H, H_{12}), 6.86 (s, 2H, H_{23}), 6.63-6.64 (t, 2H, H_{22}), 6.55-6.57 (d, 4H, H_{17}), 6.46-6.48 (m, 6H, H_{16}, H_{20}), 3.59 (s, 4H, amine proton). ^{13}C NMR (150 MHz, $CDCl_3$), δ_C (ppm): 113.9 (C_{22}), 116.3(C_{20}), 121.1 (C_{12}, C_{14}, C_{17}), 124.24 (C_9), 125.9 (C_3), 126.1 (C_6, C_7, C_8), 126.3 (C_2), 127.1 (C_{24}), 128.3 (C_4), 128.9 (C_{13}), 129.5 (C_{16}), 130.8 (C_5), 131.3 (C_{23}), 132.6 (C_{10}), 135.2 (C_{18}),

141.4 (C_{19}), 143.4 (C_1), 145.1 (C_{15}), 146.1 (C_{21}), 148.5 (C_{11}).

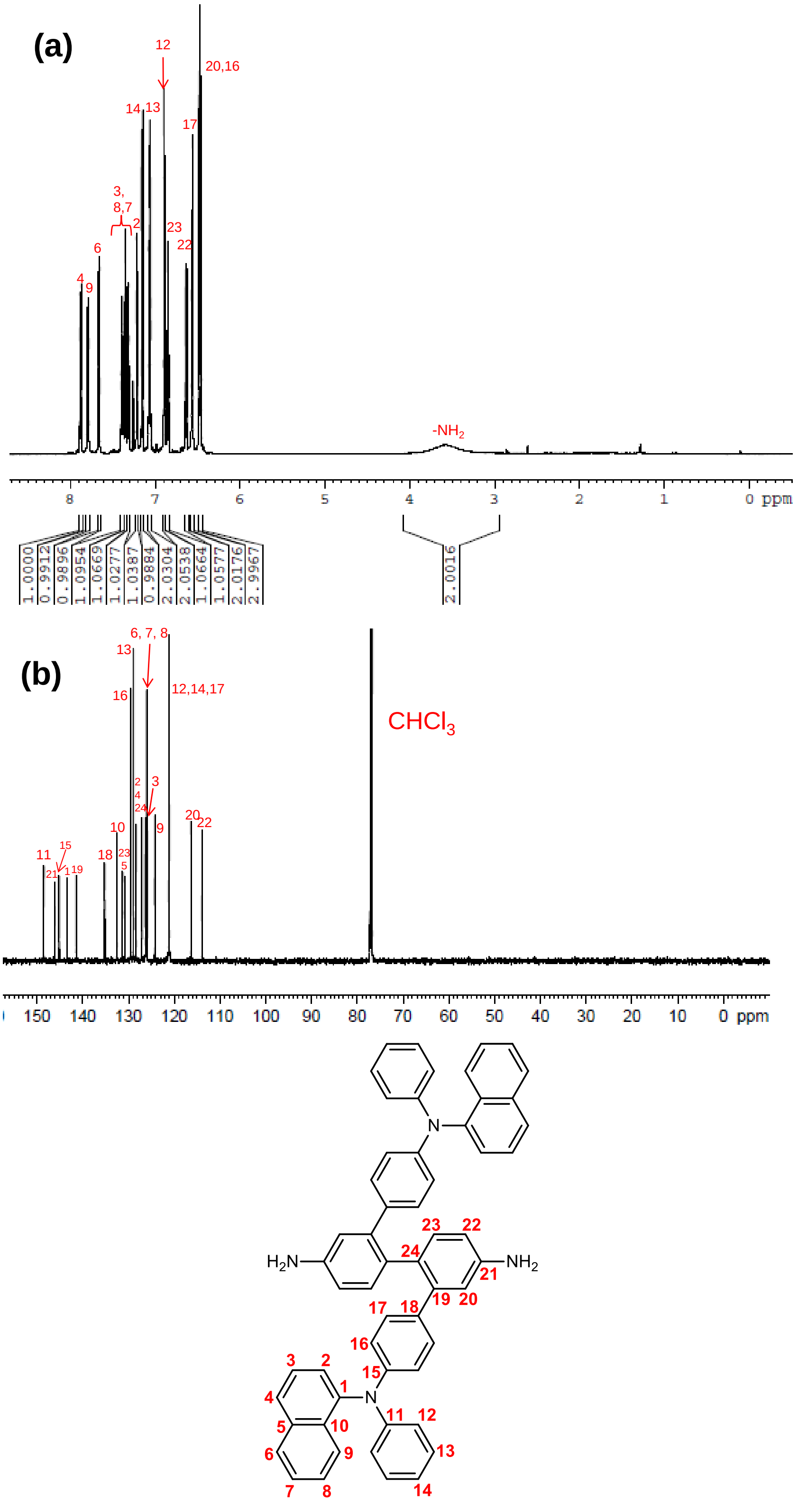

Supplementary Figure 9. NMR spectra of monomer (2). **(a)** ^{1}H NMR and **(b)** ^{13}C NMR.

Synthesis of compound PI-1a

To a stirred solution of 0.39 g (0.5 mmole) of Monomer (2) in 5 mL of *N*-methyl-2-pyrrolidinone (NMP), 0.23 g (0.5 mmole) of Monomer (a) was gradually added. The mixture was stirred at ambient temperature for 8 h to form the poly(amic acid). Chemical cyclodehydration was carried out by addition of 1 mL of acetic anhydride and 0.5 mL of pyridine into the above-mentioned poly(amic acid) solution with stirring at room temperature for 1 h, and then heating at 130 °C for 2 h. Then the polymer solution was poured into methanol. The precipitate was filtered, washed with methanol, and dried at 100 °C under vacuum to obtain PI-1a (0.59 g, 98%) as white solid. ^{1}H NMR (600 MHz, DMSO-d_6), δ_H (ppm): δ 9.85 (s, 2H, H_{36}), 7.88 (s, 2H, H_4), 7.77 (s, 2H, H_6), 7.70 (s, 2H, H_9), 7.51-7.55 (m, 6H, H_{23} and H_{35}), 7.41 (s, 4H, H_7 and H_3), 7.34 (s, 4H, H_{22} and H_8), 7.19 (s, 2H, H_2), 7.13-7.15 (t, 2H, H_{20}), 7.08 (s, 4H, H_{13}), 6.85 (s, 2H, H_{14}), 6.79 (s, 4H, H_{12}), 6.41-6.45 (d, 8H, , H_{16} and H_{17}), 3.27 (d, 2H, H_{26}), 3.09 (s, 2H, H_{25}), 2.41–2.47 (d, 2H, H_{28}), 1.44-2.19 (12H, H_{27}, H_{29} and H_{30}). ^{13}C NMR (150 MHz, DMSO-d_6), δ_C (ppm): 20.3 (C_{27C}), 23.7, 23.9 (C_{27T} and C_{30T}), 24.7, 24.8 (C_{29T} and C_{29C}), 28.3 (C_{30C}), 39.0 (C_{25}, C_{26} and C_{28}), 119.5 (C_{35}), 120.3 (C_{17}), 120.8 (C_{12}), 121.5 (C_{14}), 123.2 (C_9), 125.1 (C_{22}), 126.1 (C_7), 126.3 (C_3 andC_8), 126.7 (C_6), 127.0 (C_2), 127.3 (C_{20}), 128.4 (C_4), 129.1 (C_{13}), 129.3 (C_{16}), 130.4 (C_5), 131.7 (C_{23}), 131.9 (C_{24}), 132.7 (C_{19}), 134.5 (C_{34}), 134.7 (C_{10}), 138.2 (C_{21}), 140.3 (C_{18}), 142.2 (C_1), 146.1 (C_{15}), 147.4 (C_{11}), 172.8, 173.0 (C_{33}), 177.8, 178.1 (C_{31} and C_{32}). IR (KBr): 3398 cm^{-1} (amide N-H stretch), 3035 cm^{-1} (sp^2 C-H stretch), 2939 cm^{-1} & 2868 cm^{-1} (sp^3 C-H stretch), 1668 cm^{-1} (amide C=O stretch), 1593 & 1477 cm^{-1} (C=C aromatic stretch), 835 cm^{-1} (aromatic C-H out of plane). PI-1a exhibited a T_g of 273 °C and a T_{d10} of 456 °C under nitrogen atmosphere. PI-1a showed a inherent viscosity of 1.85, measured in NMP at a concentration of 0.5 g dL^{-1} at 30 °C.

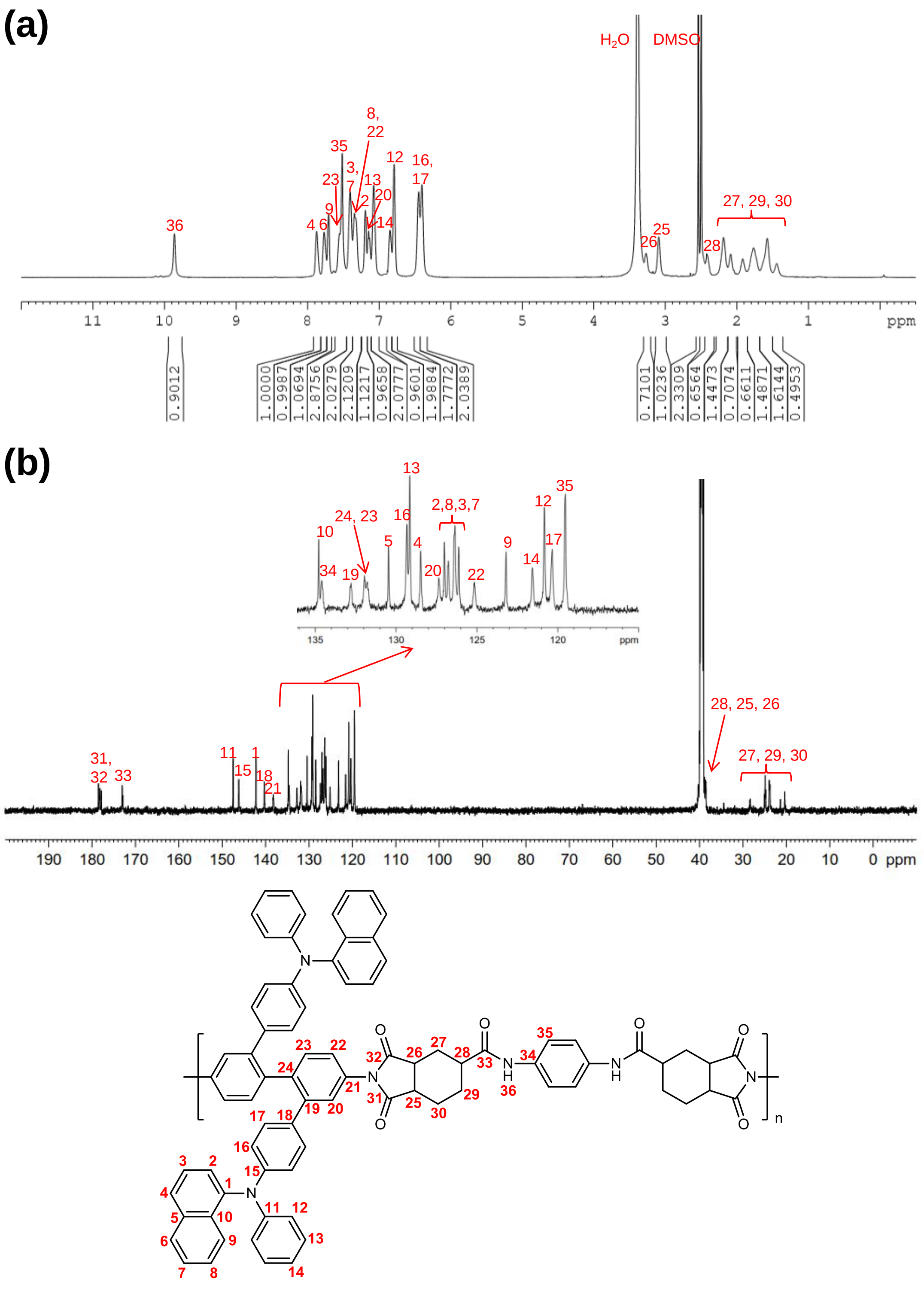

Supplementary Figure 10. NMR spectra of PI-1a. **(a)** ^{1}H NMR and **(b)** ^{13}C NMR.

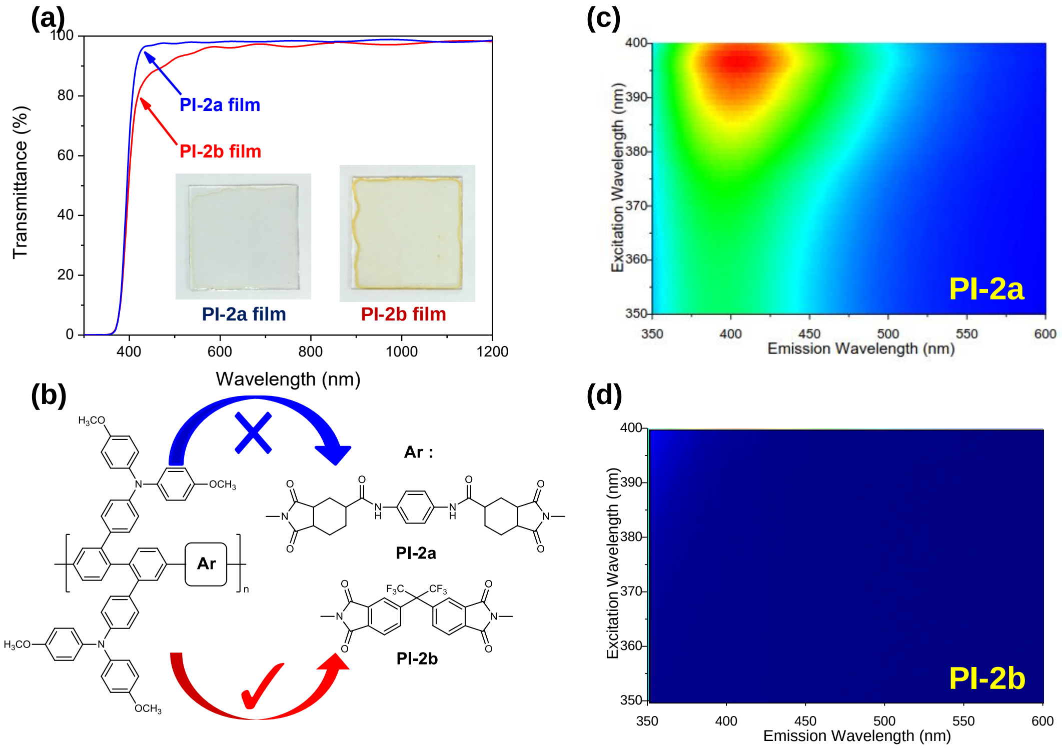

Supplementary Figure 11. Optical spectra of polyimides. **(a)** Transmittance of PI-2a and PI-2b **(b)** chemical structures of PI-2a and PI-2b, "×" means no charge transfer, "✔" means charge transfer **(c)** and **(d)** photoluminescence excitation of PI-2a and PI-2b films.

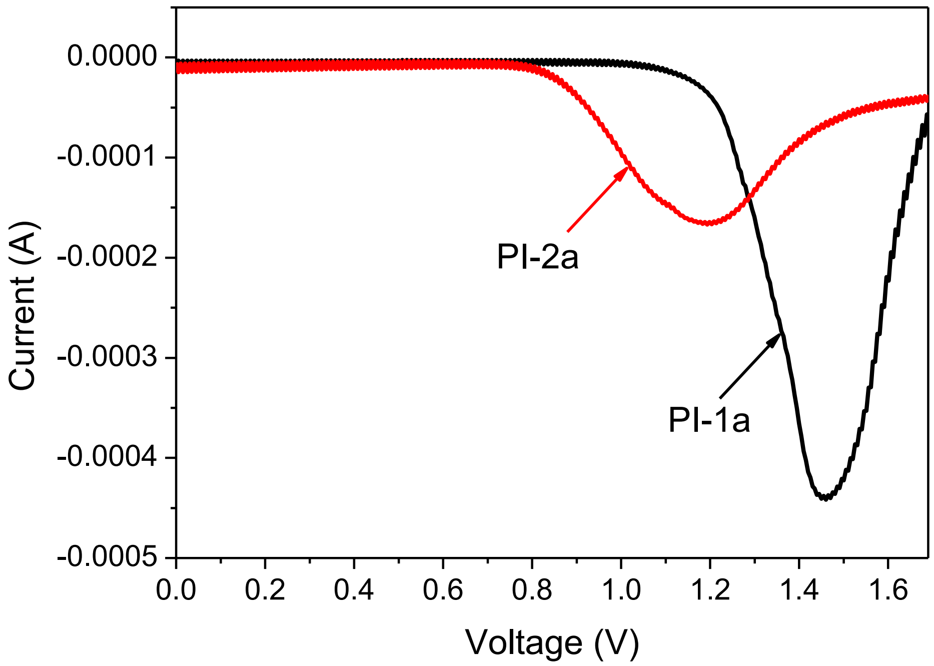

Supplementary Figure 12. DPV data of PI-1a and PI-2a films. No significant electron coupling could be observed both in PI-1a (black curve) and PI-2a (red curve) films.

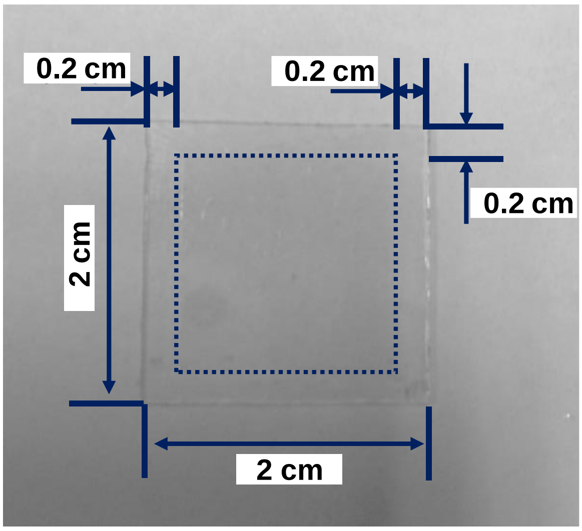

Supplementary Figure 13. PI-1a film by cutting off the edge. The thick edge (0.2 cm width) of five pieces of polymer films were cut off and the total resulting edge was weight for the calculation of film density.

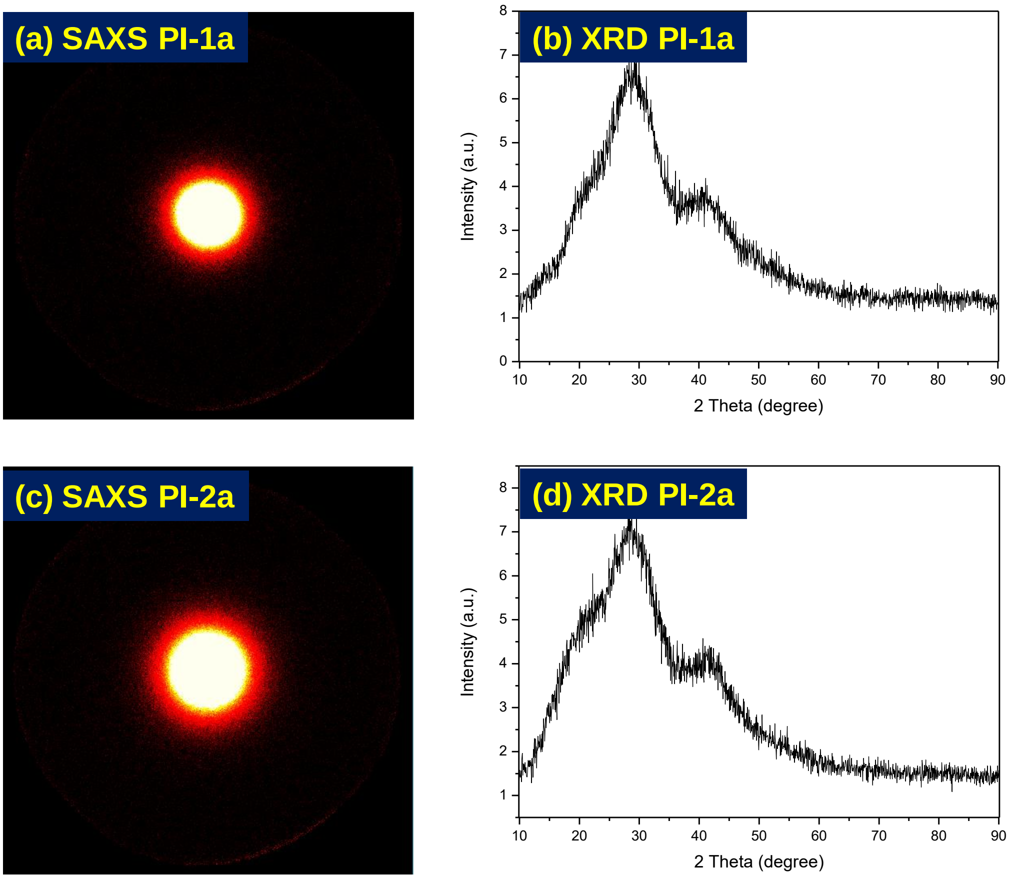

Supplementary Figure 14. SAXS and XRD data of PI-1a and PI-2a films. **(a)** SAXS of PI-1a. **(b)** XRD of PI-1a. **(c)** SAXS of PI-2a. **(d)** XRD of PI-2a. The SAXS and XRD spectra indicated both PI-1a and PI-2a films were amorphous.

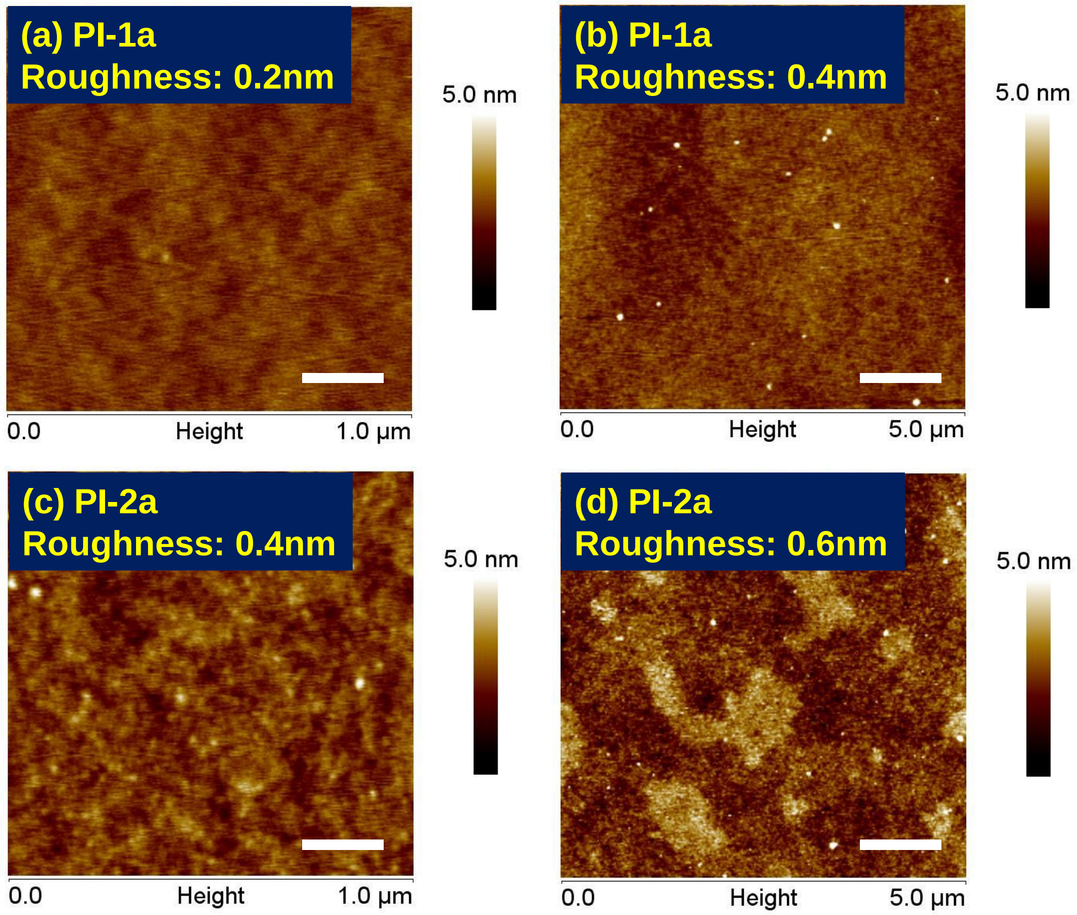

Supplementary Figure 15. AFM of PI-1a and PI-2a films. **(a)** PI-1a film. The scale bar is 200 nm. **(b)** PI-1a film. The scale bar is 1 μm. **(c)** PI-2a film. The scale bar is 200 nm. **(d)** PI-2a film. The scale bar is 1 μm.

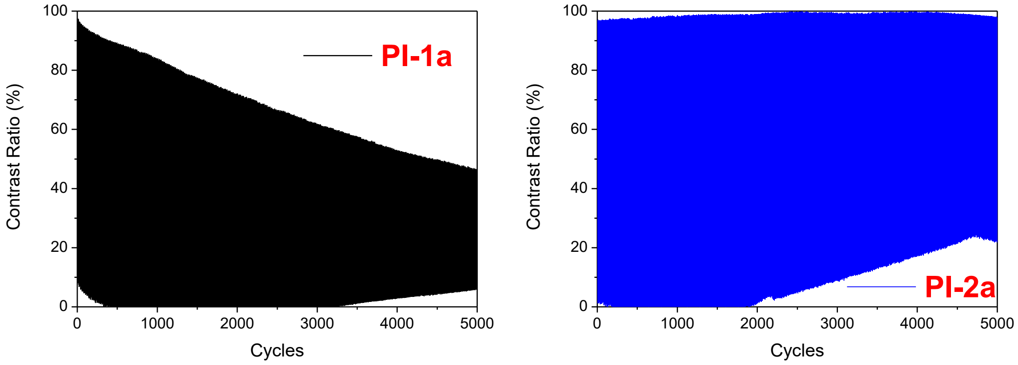

Supplementary Figure 16. Stability of PI-1a and PI-2a.

Stability of PI-1a (between 0 V and 1.3 V) and PI-2a (between 0 V and 1.2 V) films over 5000 cycles. The higher stability of PI-2a film than that of PI-1a was attributed to the methoxy group, which prevented the intermolecular coupling reaction.[7]

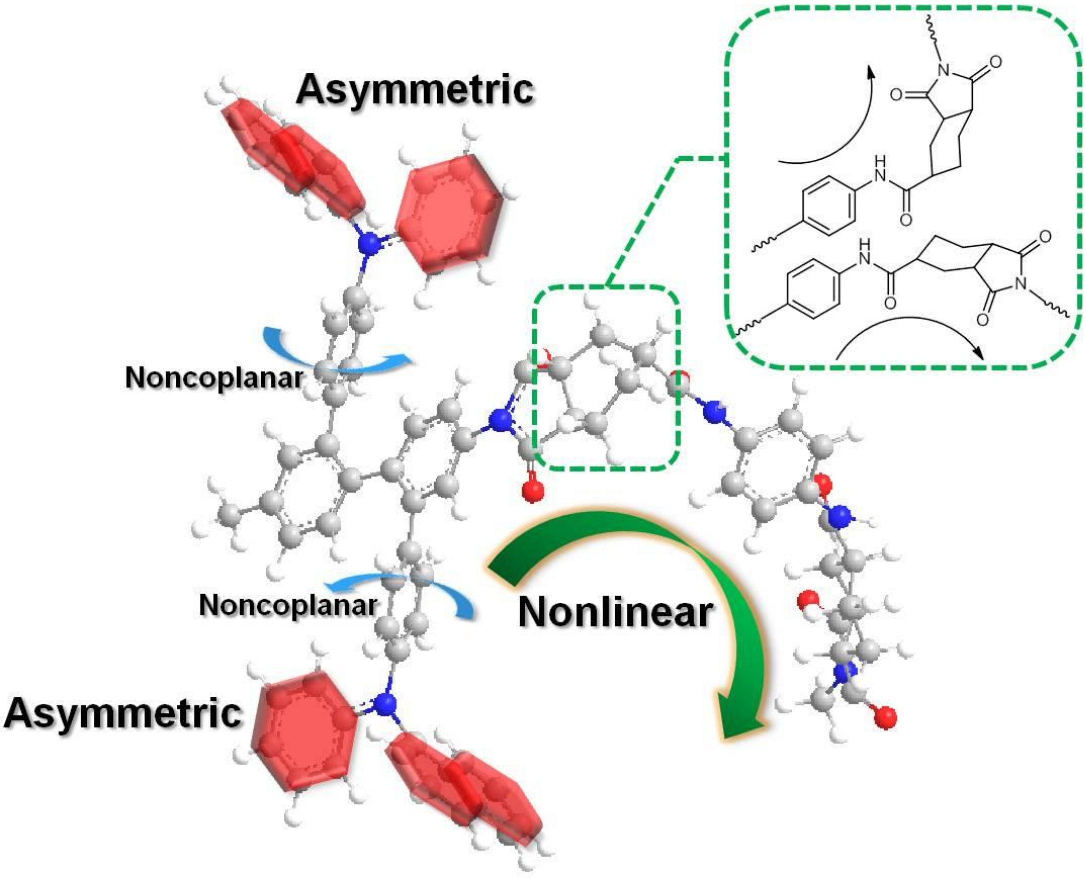

Supplementary Figure 17. Twisted structure of PI-1a.

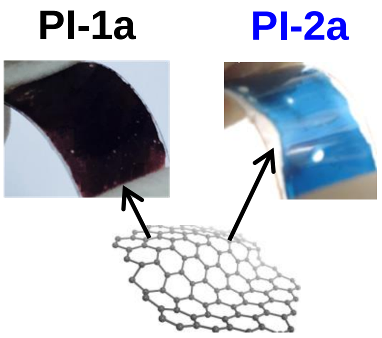

Supplementary Figure 18. EC colors of PI-1a and PI-2a films on graphene/PET films.

Supplementary Table 1. The optical and electrochemical properties.

Polymer	λ_{max} (nm)	λ_{onset} (nm)	E_g^{opt} (eV)	E_{onset} (V)	*HOMO* (eV)	*LUMO* (eV)
PI-1a	328	400	3.1	1.0	-5.4	-2.3
PI-2a	340	390	3.2	0.9	-5.3	-2.1

Supplementary Table 2. The switching time and bleaching time.

Polymer	Switching time (s)	Bleaching time (s)
PI-1a	1.3	1.1
PI-2a	2.2	3.7

Supplementary Table 3. The coordinate points.

	$L*$ (PI-2a)	$a*$ (PI-2a)	$b*$ (PI-2a)	$L*$ (PI-1a)	$a*$ (PI-1a)	$b*$ (PI-1a)
0 V	99.1	-3.5	0.2	99.7	0.2	1.0
0.7 V	99.1	-3.5	0.2	-	-	-
0.75 V	98.5	-5.3	-0.7	-	-	-
0.8 V	97.3	-5.5	-3.0	99.7	0.1	0.9
0.85 V	93.1	-8.8	-7.9	99.1	0.1	0.9
0.9 V	85.4	-13.3	-14.9	97.9	0.1	0.9
0.95 V	74.5	-17.8	-19.9	93.7	0.1	0.6
1 V	63.1	-19.4	-20.4	86.0	-0.3	-1.1
1.05 V	55.9	-19.1	-19.2	75.0	-0.3	-1.5
1.1 V	54.0	-18.6	-18.9	56.7	-0.3	-1.4
1.15 V	53.8	-18.4	-18.8	38.5	-0.4	-0.7
1.2 V	53.9	-18.5	-18.9	30.2	-1.2	-0.3

1.25 V	53.9	-18.0	-18.7	22.9	-2.1	-0.5
1.3 V	-	-	-	21.0	-2.1	-0.7
1.35 V	-	-	-	20.3	-1.7	-0.7

Supplementary References

1. Talipov, M. R., Hossain, M. M., Boddeda, A., Thakur, K. & Rathore, R., A search for blues brothers: X-ray crystallographic/spectroscopic characterization of the tetraarylbenzidine cation radical as a product of aging of solid magic blue. *Org. Biomol. Chem.* **14**, 2961–2968 (2016).

2. Christoph, L. et al. Photoinduced Charge Transfer Processes along Triarylamine Redox Cascades. *J. Am. Chem. Soc.* **127**, 10600-10610 (2005).

3. Lumpi, D. et al. Substituted triphenylamines as building blocks for star shaped organic electronic materials. *New J. Chem.* **39**, 1840-1851 (2015).

4. Shin, D.-W. et al. U.S. Pat. Appl. Publ. 26 pp. US 2007276160 (2007).

5. Loan, P. T. K. et. al. Graphene/MoS_2 heterostructures for ultrasensitive detection of DNA hybridisation. *Adv. Mater.* **26**, 4838-4844 (2014).

6. Docherty, C. J. et. al. Extreme sensitivity of graphene photoconductivity to environmental gases. *Nat. Commun*. **3**, 1228 (2012).

7. Liou, G.-S. & Chang, C.-W. High stable anodic electrochromic aromatic polyamides containing N,NN',N',-tetrahenyl-p-phenylenediamine moieties: synthesis electrochemical, and electrochromic properties. *Macromolecules* **41**, 1667-1674 (2008).
